# DNA barcoding unmasks overlooked diversity improving knowledge on the composition and origins of the Churchill algal flora

**DOI:** 10.1186/1472-6785-13-9

**Published:** 2013-03-16

**Authors:** Gary W Saunders, Daniel C McDevit

**Affiliations:** 1 Centre for Environmental and Molecular Algal Research, Department of Biology, University of New Brunswick, Fredericton, NB E3B 5A3, Canada

**Keywords:** Arctic, Barcoding biotas, DNA barcoding, Floristic survey, Macroalgae, Phaeophyceae, Rhodophyta, Thermogeographic model, Trans-Arctic exchange, Ulvophyceae

## Abstract

**Background:**

Sampling expeditions to Churchill in the Canadian subarctic were completed with the aim of compiling a molecular-assisted survey of the macroalgal flora (seaweeds) for comparison to published accounts for this area, which are based on morphological identifications. Further, because the Churchill region was covered by ice until recently (~10,000 before present), the current algal flora has had to migrate from adjacent waters into that region. We used our DNA barcode data to predict the relative contribution of the North Atlantic and North Pacific floras (Likely Source Region) in repopulating the Churchill region following the most recent glacial retreat.

**Results:**

We processed 422 collections representing ~50 morpho-species, which is the approximate number reported for this region, and generated DNA barcode data for 346 of these. In contrast to the morpho-species count, we recovered 57 genetic groups indicating overlooked species (this despite failing to generate barcode data for six of the ~50 morpho-species). However, we additionally uncovered numerous inconsistencies between the species that are currently listed in the Churchill flora (again as a result of overlooked species diversity, but combined with taxonomic confusion) and those identified following our molecular analyses including eight new records and another 17 genetic complexes in need of further study. Based on a comparison of DNA barcode data from the Churchill flora to collections from the contiguous Atlantic and Pacific floras we estimate that minimally 21% (possibly as much as 44%) of the Churchill flora was established by migration from the Pacific region with the balance of species arriving from the Atlantic (predominantly North American populations) following the last glacial retreat.

**Conclusions:**

Owing to difficulties associated with the morphological identification of macroalgae, our results indicate that current comprehension of the Canadian Arctic flora is weak. We consider that morphology-based field-identifications are ill-advised in carrying out floristic and ecological surveys for macroalgae and that much of the current data, at least for the Canadian Arctic, should be used with caution. Our efforts to use DNA barcode data to identify the most Likely Source Regions for macroalgal species currently found in Churchill suggests that migration from both the Atlantic and the Pacific have played important roles in establishing the Canadian Arctic flora. This result has significance for understanding both the current and future biodiversity and biogeography of macroalgae in these waters.

## Background

In 1883 Kjellman [1] described the Arctic seaweed flora under three broad themes: ‘scarcity of individuals’ owing to the patchy nature of suitable habitat (although dense beds are found in suitable areas); ‘monotony’ referring apparently to the dull appearance of the flora owing to the dominance of a few laminarialean species (kelp); and ‘luxuriance’ in reference to the massive size attained by some of the kelps in this area. These descriptors become exaggerated in the Churchill region, which, although essentially subarctic, acquired its post-glacial algal flora as a result of species migration from the Atlantic and Pacific Oceans through the contiguous waters of the Canadian Arctic. In summarizing floristic work at the time, Taylor [2] stated that the “Hudson Bay area is little known, but from the three or four papers which describe its flora we may judge that … we have a relatively poor flora of subarctic and arctic types.” More recent studies in Hudson Bay in general, and near Churchill in particular, have done little to change this generalization [3,4].

This paucity of diversity is not overly surprising considering both the paleoecology and contemporary ecology of the area [2,5]. For the former, this region was largely covered by sea ice until ~10,000 years ago [6] meaning there was limited if any suitable habitat for marine macroalgal growth in the region until relatively recently. As a result, the Churchill algal flora has had to reestablish from essentially Atlantic and/or Pacific source populations during subsequent warming. From a contemporary ecological perspective, ice scouring limits the intertidal growth in many areas to ephemeral annuals, while longer-lived species are confined to the subtidal [2,7]. Further, the dark winter, typical ice and snow cover, and the oblique angle of solar illumination in the summer all serve to limit the light available for photosynthesis and thus algal abundance, productivity and diversity [8].

**Figure 1:**
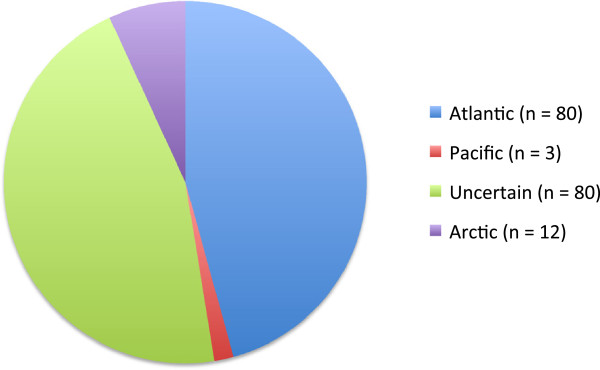
**Distribution of Canadian Arctic seaweeds in three oceans as modified from Lee** [[9]]**.** Species reported by Lee as occurring in both the Atlantic and Pacific were recorded as uncertain for comparison to the LSR summaries generated here. Arctic refers to 12 species that were considered to be largely Arctic endemics (for the purposes of the LSR summary here, they still had to migrate into the Churchill region from a source population following the most recent glaciation).

There has been a persistent view in phycological literature that the eastern Canadian Arctic flora is essentially a depauperate range extension of North Atlantic species in common to the Atlantic Provinces of Canada and northern New England with a few so-called Arctic component species [2,9-11], most of which are nonetheless also found in waters around the Atlantic Provinces of Canada [11]. Taylor [2] considered that “the western species hardly reach as far east as Hudson Bay” owing to the general paucity of suitable solid substratum west of Hudson Bay and the prolonged persistence of the ice cover in that region of the Canadian Arctic, which both reduces light for photosynthesis and deters vertical mixing of water presumably limiting nutrient availability [3,8,9]. Lee [9] presented this pattern graphically reporting that of the ~175 seaweed taxa reported in the Canadian Arctic, 80 were shared in common to only the Atlantic, another 80 in common to the Atlantic and Pacific floras, only three exclusively in common with the Pacific and 12 considered as Arctic endemics (Figure 1). In presenting these observations, Lee [9] attributed this pattern to the “wide-open communication between the Atlantic and arctic waters,” in addition to the westward barriers discussed above. However, Lee [9] also noted that the “continuous north-ward flow of Pacific water through the narrow Bering Strait should not be overlooked, for this intrusion occurs in a great volume.”

Despite this tendency to relate contemporary Arctic repopulation more closely to the Atlantic in the floristic literature [2,9-11], some of the iconic Arctic species are considered to have their evolutionary origins in the Pacific [12] as part of the “Great Trans-Arctic Biotic Interchange” [13], which initiated about 3.5 mya with the opening of the Bering Strait. During this period the invertebrate fauna of the Canadian Arctic and North Atlantic was radically impacted by a flood of Pacific species [13,14]. As argued in Adey et al. [12], and references therein, this phenomenon was also a significant contributor to the cold-tolerant algal flora of the Arctic, subarctic and North American North Atlantic (see Cánovas et al. [15] for a detailed example in the brown algal family Fucaceae for which the North Atlantic genera and species derived from four independent Pacific to Atlantic trans-Arctic colonization events). This initial period of westerly migration would have been intermittently interrupted by Pleistocene glaciation resulting in vicariant population isolation [6,14,16], which should leave a signature in variable molecular markers such as the cytochrome c oxidase 1 (COI-5P) as has been observed in marine invertebrate groups (e.g., [17,18]). The intermittent phylogeographical origins of the Canadian Arctic algal species [12] should thus have left a contemporary signal by which to establish the recent re-colonization of the Churchill region from the two source regions. Adey et al. [12] argue, based on the Thermogeographic Model, that for benthic seaweeds at least, the Trans-Arctic Biotic Exchange is a pattern that has continued to impact the composition of the Canadian Arctic and North Atlantic floras throughout the Pleistocene, as has also been posited for some invertebrate species (e.g., [19]). This would mean that not only do species in the Canadian Arctic have their evolutionary histories (phylogeography) in the North Pacific region, but that the colonization of these waters subsequent to the last glacial retreat should also have a strong Pacific component [5] in contrast to the view common in the floristic literature that colonization has been largely from the North Atlantic (e.g., [2,9-11]).

The objectives of this paper were twofold. First, to augment the detailed morphology-based species compilations for the Canadian Arctic of Lee [3] and other researchers (e.g., [4]) with molecular species identifications to assess the accuracy of current floristic accounts in light of confounding factors such as phenotypic plasticity and overlooked diversity, which are known to hinder accurate species determinations for seaweeds (e.g., [20,21]). As it has been speculated that the current warming trend will facilitate a renewed wave of migration of Pacific marine species into the warming Canadian Arctic (a contemporary “Arctic Invasion”) and eventually into the cold-temperate North Atlantic [14], a survey of the algal flora from the Churchill region to establish a contemporary baseline for future comparisons is particularly timely. Second, to extend the results of the first objective to consider the source regions for the algal species that have re-colonized the Churchill flora (and by extension the Canadian Arctic flora) since the last glacial maximum. As discussed above, the most detailed survey of the Canadian Arctic flora to date concluded that re-colonization had been predominantly from the Atlantic with only three species or 1.7% (Figure 1) considered to have unequivocally re-colonized from the Pacific [3]. Critically, this conclusion was based on morphological species identifications and a relatively poor comprehension of the North Pacific subarctic flora, both factors that can strongly bias interpretations on the origins and migrations of algal taxa [12,16]. Further, this perspective is at odds with biogeographical patterns established for the Canadian Arctic and North Atlantic in the animal and more recent algal literature [5,12,13,17,19].

## Methods

### Field methods

As partners in a project to DNA barcode the Churchill biota, we were able to collect marine algal samples from August 19–25, 2006 (n = 201) and July 7–13, 2007 (n = 221). Samples were collected both in the intertidal and subtidally by scuba to 18 m. Every effort was made to maximize the number of species obtained and to acquire multiple collections for each species when possible. In the laboratory, each specimen was field-identified (based largely on Sears [11]), photographed, pressed as a herbarium voucher, and a small amount placed into silica gel for subsequent molecular analyses.

New records for Hudson Bay and the Canadian Arctic are relative to the detailed geographical data in Lee [3] and the recent account of Mathieson et al. [4], and those listed for the Atlantic and Pacific regions to Sears [11] and Gabrielson et al. [22], respectively.

### Molecular methods

All molecular methods are outlined in detail in Saunders & McDevit [23]. For the green algae (Chlorophyta) the markers *tufA* and or *rbc*L-3P were used to generate the barcode data [24], while for brown (Phaeophyceae; [21]) and red (Rhodophyta; [25]) algae the marker COI-5P was used. The actual primer pair to amplify the respective barcode marker from each collection is recorded with that entry on both GenBank and BOLD (http://www.boldsystems.org/). Data generated in this study for collections from Churchill were supplemented with data from BOLD (not listed in Additional file 1) for conspecific and congeneric collections from the Atlantic (predominantly North America) and Pacific (predominantly British Columbia) regions. Sequence data were used to generate cluster-based trees using Jukes & Cantor distances with UPGMA in the program Geneious Pro 5.6.3 [26] to assign collections to genetic species groups. In many cases (Results) collections traditionally assigned to accepted morpho-species were resolved as multiple genetic groups indicating within-species population structure or the presence of overlooked species. When the previous genetic divergence was associated with geography distinguishing between the Atlantic and Pacific region floras, with our Churchill collections typically resolving within one of these genetic groups, then the data were used to predict a source region (population) for recolonization of the Churchill flora following the last glacial retreat (herein termed the Likely Source Region).

### Likely source region summary

Only those species for which barcode data were generated are presented here (Additional file 1). In our Likely Source Region predictions, unless otherwise indicated, regions are referred to as follows: Arctic region – the Canadian subarctic and Arctic only; Atlantic region – the North American Atlantic (the few European collections indicated where necessary); and Pacific region – broad sense including north Pacific, Bering Sea, Chukchi Sea, etc. (i.e., the likely source for the Churchill populations is considered to be from a western source). By ‘Likely Source Region’ we are strictly trying to ascertain if a species currently inhabiting the Churchill region migrated there from the Atlantic or the Pacific region following the most recent glacial retreat.

## Results

### Barcode survey of Churchill flora

Two collection excursions to Churchill during August 2006 and July 2007 resulted in 422 macroalgal specimens from intertidal and subtidal habitats. These were roughly assigned to ~50 morpho-species during field identifications, although in many cases with some uncertainty and with some specimens left unidentified. This compares well to the recent summary of species in James Bay by Mathieson et al. [4] for which 44 morpho-species were listed. We managed to generate barcode records for 346 of these collections – 15 were Ulvophyceae (13 *tufA* and 12 *rbc*L-3P barcodes generated), 187 Phaeophyceae (COI-5P) and 144 were Rhodophyta (COI-5P) – resulting in 57 genetic species groups (Additional file 1). We were unable to generate barcode data for the collected Cladophorales, all of which were morphologically identified as *Chaetomorpha melagonium* (F. Weber & D. Mohr) Kützing (n = 3), which is typical for this group of green algae [24]. Additionally, representative specimens of the brown algal taxa *Ralfsia* sp. (n = 5), *Sphacelaria artica* Harvey (n = 2) and *Sphacelaria plumosa* Lyngbye (n = 12) and the red algal taxa *Hildenbrandia* sp. (n = 1) and *Turnerella pennyi* (Harvey) F. Schmitz (n = 1) were not successfully barcoded with the primers used during this study. These collectively account for six (of the ~ 50) morpho-species in need of further study in the Churchill flora. In addition to overlooked species, which accounted for much of the discrepancy between our morphological (~50) and molecular (57) species tallies, we uncovered a number of taxonomic anomalies between the species that are currently listed as present in the Canadian Arctic [3,4,11] and the species we actually identified following our molecular analyses (discussed below).

### Likely source region (LSR) summary

**Figure 2:**
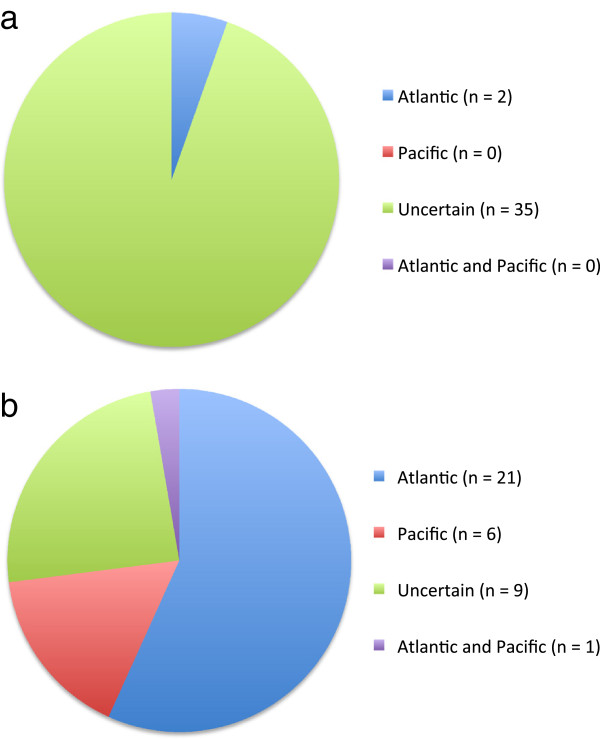
**LSR summaries for Churchill populations following our barcode survey. a**) Predicted source region based on morphology of the genetic species groups. **b**) Predicted source region based on molecular data.

Figure 1 depicts the relative distribution for ~175 morpho-species reported in the Canadian Arctic compared to the Atlantic and Pacific regions as compiled by Lee [9]. This Figure was used to support the widely held notion of the time that the Canadian Arctic flora is essentially a depauperate extension of the Canadian North Atlantic flora (minimally 45.7%) with a very minor uniquely Pacific component (1.7%). An additional 45.7% of the morpho-species reportedly occurred in both the Atlantic and Pacific regions and thus attempts to identify a source region were not possible (LSR uncertain) for this component of the flora (Figure 1). This summary is strictly based on morphological identifications and included all of the Arctic specimens that Lee [9] could verify to that point in time. Here we use our DNA-barcode analyses of the Churchill flora (including a few collections from a trip to the northern tip of Baffin Island, Nunavut, where applicable) to complete contemporary morphological and molecular comparisons to Lee’s [3] detailed morpho-species summary to determine how accurately his summary reflects the origins of the Canadian Arctic flora (Figure 2).

Of the 57 genetic groups from Churchill for which barcode data were available (Additional file 1), we were able to include 37 (65%) in our Likely Source Region (LSR) summary (Figure 2). The remaining 20 were excluded largely owing to a lack of specimens for the corresponding morpho-species from the Pacific region (detailed in the following section). We first predicted an LSR for each of these 37 species based on morphology (field identification) recording ‘uncertain’ when the individuals of a species were found in both the Atlantic and Pacific source regions or could be assigned to more than one genetic group in either (or both) of these source regions. Interestingly, adhering to the previous criteria, we could only assign an LSR to two of the 37 species (~5%) both being Atlantic with ~95% of the included species having an uncertain LSR based solely on morphology (Figure 2a). This contrasts the summary in Lee ([3]; in the following calculations excluding his ‘Arctic morpho-species’ as he presented the data from a distributional rather than source region perspective) for which the uncertain component would apply to ~49% of the flora (Atlantic and Pacific distribution), with ~49% likely Atlantic and only ~2% likely Pacific in origin (Figure 1). The discrepancies between Lee’s percentages and those determined here can be attributed to greater phenotypic plasticity for some species then previously appreciated (e.g., *Dictyosiphon* species complex), overlooked diversity (e.g., *Desmarestia aculeata* complex), range extensions for some species (e.g., *Pylaiella washingtoniensis*) and some genera in need of substantial taxonomic revision in the North American flora (e.g., *Phycodrys* species complex) (all detailed in the following section). In time, taxonomic research will resolve many of these issues, at least for use with characters at the anatomical level, but results here suggest that morphology-based field-identifications are ill-advised in carrying out ecological surveys for many species of seaweeds (certainly for the taxa discussed in the current manuscript).

Not surprisingly, we had far greater success in our LSR assignments using our molecular data, identifying regions for 76% of the 37 included species (Figure 2b), which exceeded both of the previous morphology based summaries. This is because molecular data circumvented the issues outlined above when dealing with morphological data (e.g., phenotypic plasticity), and also provided population level signal for some of the species reported here. Although the Atlantic still dominated as the LSR for the Churchill flora accounting for 57% of the species, our data contrast predictions for a Pacific LSR relative to Lee ([3]; estimated at only 1.7%, Figure 1) indicating that 16% of the species may have established in the Churchill flora from that region (Figure 2b). Further, molecular data have indicated that Churchill populations of *Saccharina latissima* (Linnaeus) C.E. Lane, C. Mayes, Druehl & G.W. Saunders have both Atlantic and Pacific contributions [21], a result that was not evident by morphology alone (Figure 2a). If we consider only genetic species for which a molecular LSR was predicted (exclude those listed as uncertain from the calculations), this can be extrapolated to estimate that 75% of the Churchill flora has its biogeographical source from the Atlantic region, 21% from the Pacific region and 4% from both (Figure 2b).

The following section details our genetic groups and why/how they were scored for the morphological and molecular LSR summary results (Figure 2), while at the same time providing a detailed list of the genetic groups that we encountered during our survey of the Churchill flora (Additional file 1) and highlighting taxa still in need of formal taxonomic reassessment.

### Taxonomic and likely source region notes

In the following section, ‘(n = X/X/X)’ refers to number of specimens we have barcoded for that genetic species group from the Pacific region (predominantly British Columbia), Churchill region (Additional file 1; including a few collections from the northern tip of Baffin Island, Nunavut, where indicated) and Atlantic region (predominantly North American Atlantic, but some European collections where indicated), respectively.

### Ulvophyceae

Ulotrichales, Ulotrichaceae

#### *Acrosiphonia* sp._3GWS (n = 0/2/3) (taxonomic work needed; possible new species or new record for Canadian Arctic)

**LSR: Morphology =** uncertain; **DNA barcode** = Atlantic.

**Comment**: The green algal genus *Acrosiphonia* requires taxonomic revision in Canada and we have not applied a name to this genetic group. As an example, two species are reported from the Canadian Arctic and Atlantic [11], but we have identified four genetic species from this area. As the two species reported from the Arctic [3] are recorded in both the Atlantic and Pacific Oceans [16], it is safe to conclude that an LSR based on morphology is uncertain (Figure 2a). We have two collections of this genetic species from Churchill (Additional file 1) with three additional collections from the Atlantic and no matches (contradicting the reports from the previous sentence) for any of our Pacific collections (73 barcoded for *tufA* and/or *rbc*L-3P) for this genus indicating an Atlantic LSR for this genetic group (Figure 2b).

#### *Acrosiphonia* sp._6GWS (n = 0/1/0) (taxonomic work needed; possible new species or new record for Canadian Arctic)

**LSR: Morphology =** uncertain; **DNA barcode** = uncertain.

**Comment:** See comments for *Acrosiphonia* sp._3GWS regarding a morphological LSR for this species (Figure 2a)*.* As this species has only been encountered in our Churchill collections and not among 118 barcoded collections (*tufA* and/or *rbc*L-3P) from the Atlantic and Pacific regions for this genus, its LSR based on molecular data remains uncertain (Figure 2b).

#### *Spongomorpha aeruginosa* (Linnaeus) C. Hoek (n = 0/1/7) (new record for Hudson Bay)

**LSR: Morphology =** Atlantic; **DNA barcode** = Atlantic.

**Comment:** This species is reported from the Atlantic [11] and Arctic [3], but not from the Pacific [16,22]. The Atlantic is thus the logical LSR from a morphological perspective (Figure 2a). We have barcoded a total of eight specimens with a single record for Churchill, the remainder from Connecticut to Newfoundland. The molecular data are consistent with an Atlantic LSR (Figure 2b).

#### *Ulothrix flacca* (Dillwyn) Thuret (n = 0/1/4)

**LSR: Morphology =** excluded; **DNA barcode** = excluded.

**Comment:** The green algal genus *Ulothrix* requires taxonomic revision in Canada and application of this name to this genetic group is tentative. This species is recorded in all three regions under consideration here [3,11,16,22]; it is safe to conclude that an LSR based on morphology is uncertain. We have one collection of this genetic species from Churchill (Additional file 1) with five additional collections from the Atlantic and no matches in our Pacific collections to date, indicating an Atlantic LSR to this genetic group. However, we have only generated molecular data for two Pacific collections assignable to this entire genus and we thus exclude this species from the LSR summary.

### Ulvales, Kornmanniaceae

#### *Blidingia* sp._5GWS (n = 0/1/0)

**LSR: Morphology =** excluded; **DNA barcode** = excluded.

**Comment:** This is another genus in need of taxonomic work in our flora. Although this collection resolves among the few collections (n = 14 for the entire genus) for species assignable to *Blidingia* from the Atlantic and Pacific included in our alignments, we have too few collections to draw meaningful inferences and this species is excluded from our LSR summary.

### Ulvales, Ulvaceae

#### *Ulva lactuca* Linnaeus (n = 42/4/54)

**LSR: Morphology =** uncertain; **DNA barcode** = Pacific.

**Comment:** As with other genera of green algae, *Ulva* is taxonomically complex with many species difficult to discern with any certainty [24]. As such, previous records based on morphology are as likely to be based on misidentifications as they are correct identifications. This species is reportedly widely distributed, occurring in the Arctic, Atlantic and Pacific Oceans [3,11,16,22]. Interestingly, this genetic species, despite current perspectives, may not be true *U. lactuca* and taxonomic work is needed [27]. Further, *Ulva* species have been widely introduced through human activities [28] suggesting caution regarding their inclusion in an LSR prediction. In summary, an LSR based on morphology is uncertain (Figure 2a). We have generated *tufA* data for 100 collections and there are only three differences across the alignment, two fixed in the four Churchill collections, one fixed in the Atlantic versus British Columbia and Churchill collections providing weak evidence for a Pacific LSR (Figure 2b). The two shared differences in the Churchill collections may indicate recolonization from the most northerly reaches of the Pacific region (poorly sampled here) or from a northern glacial refugium, while Europe is weakly excluded as a possible source – a collection from Ireland was a genetic match to the North American Atlantic collections. More samples from these regions are needed to explore these hypotheses.

#### *Ulva prolifera* O.F. Müller (n = 1/5/12) (taxonomic work needed; possible new species or new record for Canadian Arctic)

**LSR: Morphology =** uncertain; **DNA barcode** = Atlantic.

**Comment:** This genetic species can be particularly difficult to identify and many of our collections were given other names during field identification. It is currently reported from the Arctic, Atlantic and Pacific Oceans [3,11,16,22] and thus an LSR on morphological grounds cannot be predicted (Figure 2a). In this case *tufA* did resolve the Pacific isolate as distinct from the other collections being 1% divergent (consistent with overlooked species), while the Atlantic and Churchill collections displayed only one polymorphic site (0.1%). The LSR based on molecular data is thus Atlantic (Figure 2b).

### Phaeophyceae

Desmarestiales, Desmarestiaceae

#### *Desmarestia aculeata* (Linnaeus) J.V. Lamouroux (n = 10/12/20) (taxonomic work needed; possible new species or new record for Canadian Arctic)

**LSR: Morphology =** uncertain; **DNA barcode** = Atlantic.

**Figure 3:**
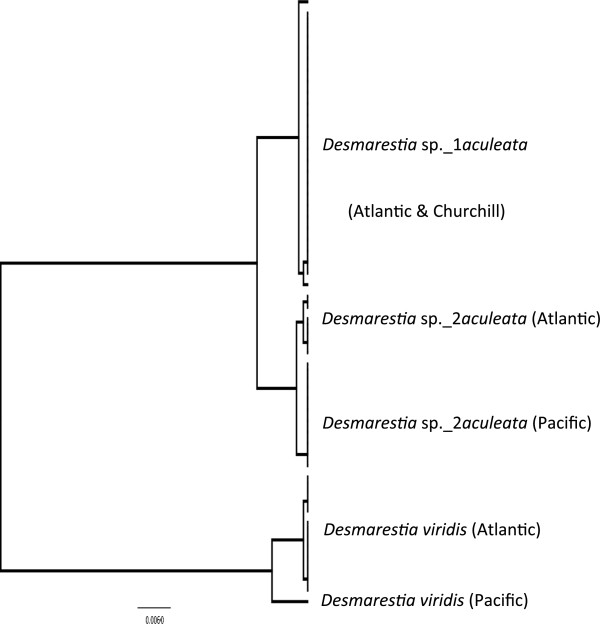
**Overlooked diversity and the biogeographical distribution of the associated mitotypes for specimens assigned to the morpho-species *Desmarestia aculeata*.
**

**Comment:***Desmarestia aculeata* is reported in all three regions under consideration here [3,11,16,22] and thus an LSR based on morphology cannot be predicted (Figure 2a). Specimens assignable to this morpho-species resolved as three genetic groups in our COI-5P analyses (Figure 3). *Desmarestia* sp._2*aculeata* had closely related Atlantic and Pacific clusters, but we did not detect it in Churchill, while *Desmarestia* sp._1*aculeata* was only in the Atlantic and Churchill (Figure 3) indicating an Atlantic LSR for this species in the Churchill flora (Figure 2b). The sp._1 and sp._2 COI-5P clusters were ~1.7-2.1% divergent, as roughly was a single isolate of *D. viridis* from British Columbia relative to its ‘conspecifics’ in the Atlantic (Figure 3; ~1.2-1.4%; this species was not encountered in our Churchill collections), consistent with an expected recent history of migration and isolation for the seaweed taxa in the regions under study here (see [12-14]).

### Ectocarpales, Acinetosporaceae

#### *Pylaiella littoralis* (Linnaeus) Kjellman (n = 7/5/23) (taxonomic work needed; possible new species or new record for Canadian Arctic)

**LSR: Morphology =** uncertain; **DNA barcode** = Atlantic.

**Figure 4:**
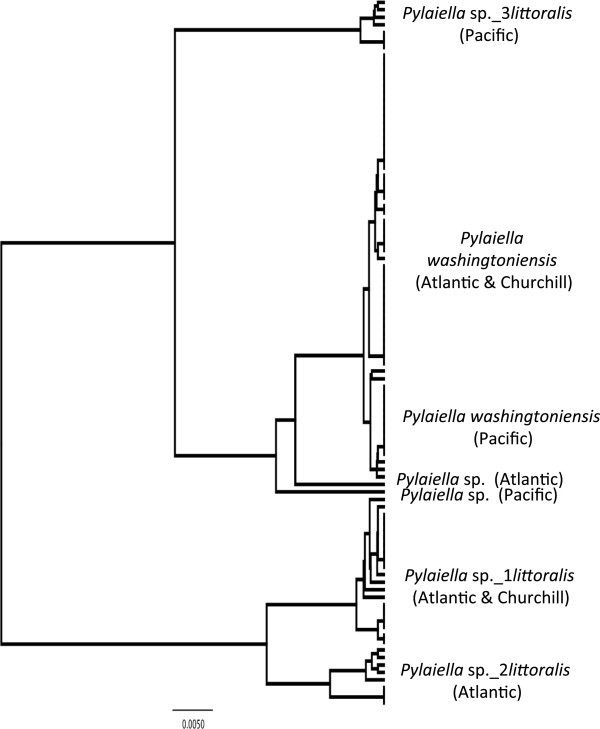
**Overlooked diversity and the biogeographical distribution of the associated mitotypes for specimens assigned to morpho-species of the genus *Pylaiella*.
**

**Comment:***Pylaiella littoralis* as a morphological species occurs in the Arctic, Atlantic and Pacific Oceans [3,11,16,22]. As discussed in Gabrielson et al. [22] this genus needs substantial taxonomic work and not even their optimism that *P. littoralis* is well defined is supported by our genetic data (Figure 4). Rather than being a single species, we have actually uncovered three genetic groups loosely assignable to this morphological species (Figure 4) and predicting an LSR on morphological grounds is thus uncertain (Figure 2a). One of these, *Pylaiella* sp._1*littoralis*, was collected only from Churchill and the Atlantic supporting the latter as the LSR for our northern populations of this morpho-species (Figure 2b).

#### *Pylaiella washingtoniensis* Jao (n = 15/16/26) (new record for Canadian Arctic & Atlantic)

**LSR: Morphology =** uncertain; **DNA barcode** = Atlantic.

**Comment:** This species is only reported from the Pacific for the regions under study here [3,11,16,22], but we have found it commonly in all three oceans (Figure 4). As this species has gone overlooked in two of the three regions under study, an LSR cannot be assessed on morphological grounds (Figure 2a). Genetically there is diversity within the species (0–0.8%) that was observed in all regions suggesting that this species has a complex history in our oceans (Figure 4). Nonetheless, the Churchill and Atlantic collections cluster together and are separated from the Pacific collections by two fixed differences indicating some past isolation and pointing to the Atlantic as the LSR for the Churchill populations (Figure 2b).

### Ectocarpales, Chordariaceae

#### *Chordaria flagelliformis* (O.F. Müller) C. Agardh (n = 4/14/52) (taxonomic work needed; possible new species or new record for Canadian Arctic)

**LSR: Morphology =** uncertain; **DNA barcode** = Atlantic.

**Figure 5:**
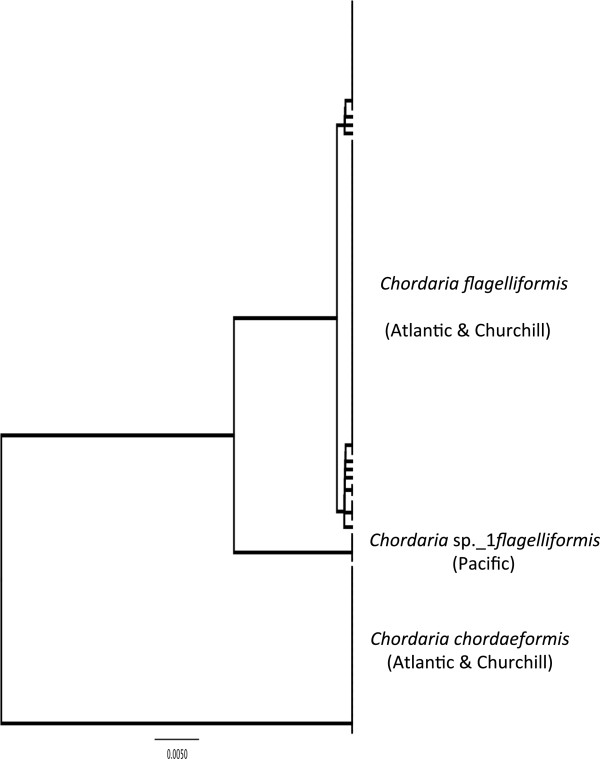
**Overlooked diversity and the biogeographical distribution of the associated mitotypes for specimens assigned to morpho-species of the genus *Chordaria*.
**

**Comment:***Chordaria flagelliformis*, the only species of this genus reported from Canada, is recorded from the Arctic, Atlantic and Pacific Oceans [3,11,16,22]. Prediction of an LSR on morphological grounds is thus uncertain (Figure 2a). Our COI-5P analyses, however, recovered two distinct genetic groups for collections assigned to this morpho-species (Figure 5), which have experienced a relatively recent isolation (~2.6-2.8% divergent) with the Churchill collections solidly of Atlantic LSR (Figure 2b). Kim & Kawai [29] also uncovered distinct Atlantic and Pacific populations for *C. flagelliformis* in their ITS analyses.

#### *Chordaria chordaeformis* (Kjellman) Kawai & S.H. Kim (n = 0/20/1) (new record for Canadian Arctic)

**LSR: Morphology =** uncertain; **DNA barcode** = Atlantic.

**Comment:** We identified a third genetic group for *Chordaria* in Canada that, based on a match to ITS data in GenBank [29], is assignable to *C. chordaeformis* (Figure 5). Kim & Kawai [29] uncovered distinct Atlantic and Pacific populations for *C. chordaeformis* (LSR based on morphology thus uncertain; Figure 2a) with our Churchill and (single) Prince Edward Island collections joining their Atlantic ITS cluster (ITS data not shown) indicating an Atlantic LSR for our Churchill collections (Figure 2b).

#### *Leptonematella fasciculata* (Reinke) P.C. Silva (n = 0/3/0)

**LSR: Morphology =** excluded; **DNA barcode** = excluded.

**Comment:** We only have three collections of this relatively obscure species all from the Churchill region, and it is thus not included in the LSR summary. This species forms miniscule brown tufts on other algae, and can be easily overlooked or ignored as it looks much like other more common brown tufts in the various floras.

### Ectocarpales, Dictyosiphonaceae

#### *Dictyosiphon foeniculaceus* (Hudson) Greville (n = 0/2/40) (taxonomic work needed; possible new species or new record for Canadian Arctic)

**LSR: Morphology =** uncertain; **DNA barcode** = Atlantic.

**Figure 6:**
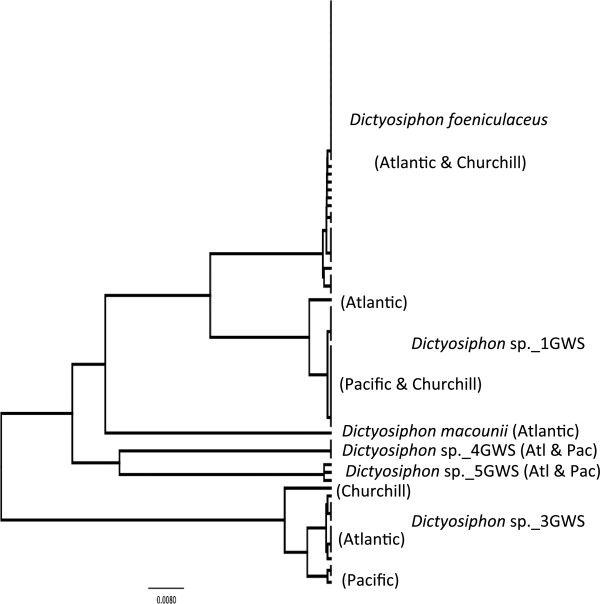
**Overlooked diversity and the biogeographical distribution of the associated mitotypes for specimens assigned to morpho-species of the genus *Dictyosiphon*.
**

**Comment:** As with many other genera, taxonomic work is required. This morpho-species is recorded from the Arctic, Atlantic and Pacific Oceans [3,11,16,22]. As such, a morphological LSR cannot be determined (Figure 2a). However, the taxonomic issues are far more complex. Whereas only four species are confidently recorded in Canada for this genus with two in common between the Atlantic and Pacific [11,22], we uncovered six genetic groups with four found in both oceans (Figure 6). Furthermore, there is typically as much morphological variation among individuals within each genetic group as there is between them such that identification based on the local floristic keys [11,22] results in nearly all morpho-species names being applied to various individuals of each genetic group. We thus only tentatively assign this genetic group to this morpho-species name. We have only recovered members of this genetic group in the Arctic and Atlantic regions, all Pacific collections falling into other genetic groups (Figure 6). The LSR thus appears to be the Atlantic (Figure 2b).

#### *Dictyosiphon* sp._1GWS (n = 15/1/1) (taxonomic work needed; possible new species or new record for Canadian Arctic)

**LSR: Morphology =** uncertain; **DNA barcode** = Pacific.

**Comment:** Owing to the comments above for *D. foeniculaceus* we consider an LSR based on morphology as uncertain (Figure 2a). This genetic group dominates in the Pacific (Figure 6) where it likely accounts for most records of *D. foeniculaceus*[22]. There are distinct COI-5P mitotypes for the lone Atlantic collection versus the Pacific collections with the Churchill collection firmly matching the latter (Figure 6) thus indicating a Pacific LSR based on molecular data (Figure 2b).

#### *Dictyosiphon* sp._3GWS (n = 3/1/13) (taxonomic work needed; possible new species or new record for Canadian Arctic)

**LSR: Morphology =** uncertain; **DNA barcode** = uncertain.

**Comment:** Owing to the comments above for *D. foeniculaceus* we consider an LSR based on morphology as uncertain (Figure 2a). There are distinct mitotypes for all three regions (Figure 6) and thus the COI-5P data are equivocal on the LSR for the Churchill populations (Figure 2b). It is possible that migration to the Churchill region in this case was from Europe, or the northern-most reaches of the Pacific region (poorly sampled here) or from a northern glacial refugium; hypotheses that can be tested with additional DNA barcode investigations of *Dictyosiphon* from these regions.

### Ectocarpales, Punctariaceae

#### *Punctaria* sp._2GWS (n = 2/3/5) (taxonomic work needed; possible new species or new record for Canadian Arctic)

**LSR: Morphology =** uncertain; **DNA barcode** = uncertain.

**Comment:** This is again a genus in need of significant taxonomic revision. This species was anatomically similar to *Punctaria plantaginea* (Roth) Greville, but differed significantly in gross morphology and COI-5P sequence. Despite being rather small and linear in outline (typically 3–20 cm long, 0.8-3 cm wide), we had hoped that these collections might represent range extensions for the cold-water species *P. glacialis* Rosenvinge, but this was not the case. Our Churchill plants have hairs, which are not known for that species [30]. Attempts to key this species out with the respective floristic guides [11,22] for the Atlantic and Pacific floras, as well as the appropriate literature cited therein, also failed to establish a name for this entity. Being apparently overlooked in all floras, an LSR based on morphology can be considered uncertain (Figure 2a). We have collections from all three regions under study here, but the COI-5P sequences are virtually identical in all collections providing no indication of population structure and suggesting a recent dispersal from one ocean basin through the Arctic to the other, but the direction of any such migration remains uncertain, as does a putative LSR based on molecular data (Figure 2b).

### Ectocarpales, Scytosiphonaceae

#### *Petalonia fascia* (O.F. Müller) Kuntze (n = 31/2/54)

**LSR: Morphology =** uncertain; **DNA barcode** = uncertain.

**Figure 7:**
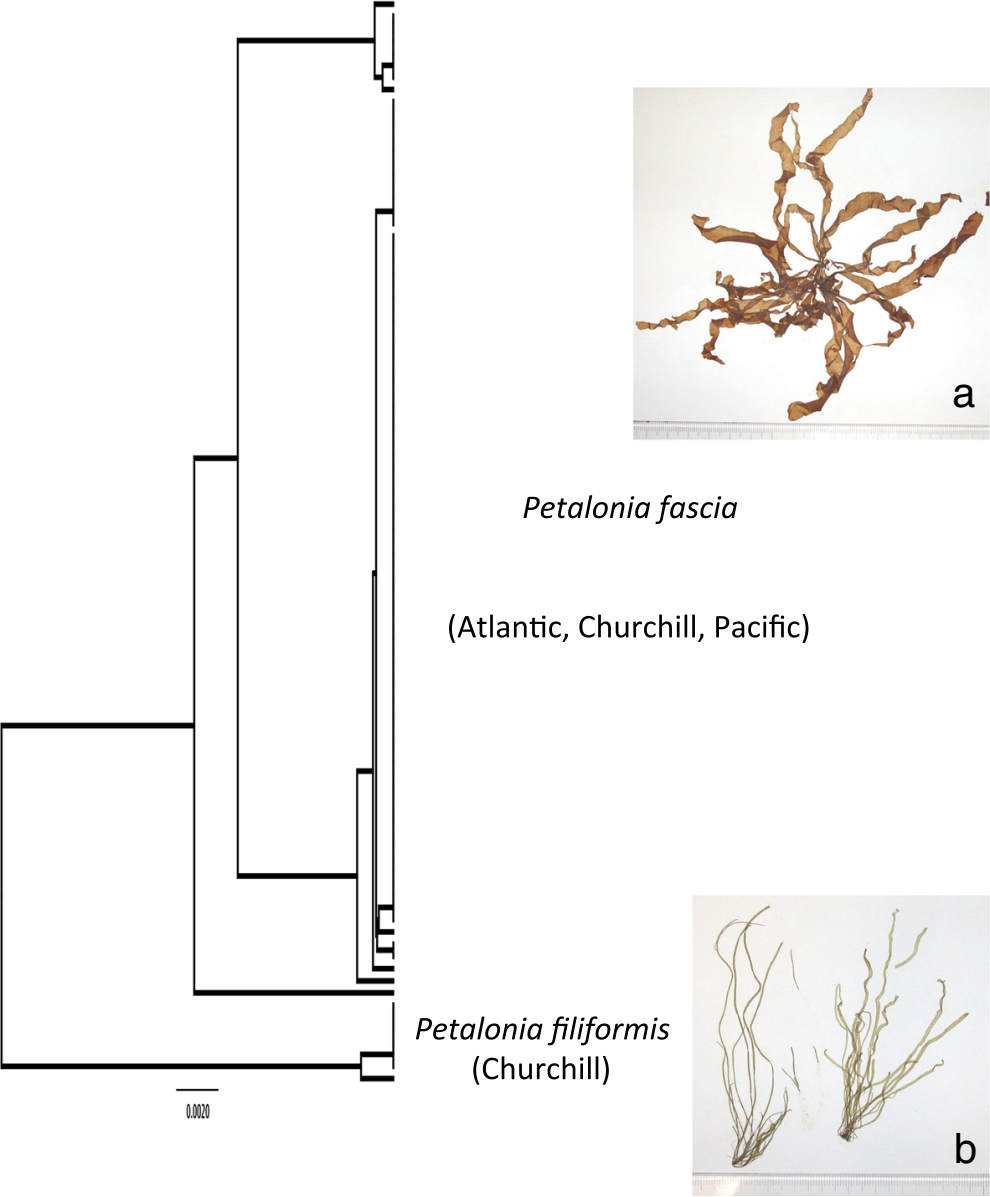
**Overlooked diversity and the biogeographical distribution of the associated mitotypes for specimens assigned to morpho-species of the genus *Petalonia*.** Includes associated images for: **a**. *P. fascia* (GWS005312); and, **b**. *P. filiformis* (GWS005259). Scale = centimeter ruler.

**Comment:** This species is reported from all three regions under study here [3,11,16,22] and thus predicting an LSR is uncertain based on morphology (Figure 2a). The COI-5P shows a few related mitotypes with Arctic collections falling in the most common type (Figure 7), which also includes collections broadly throughout the Pacific and Atlantic regions. Our ITS data (not shown) match a population in Kogame et al. [31] that was also found in the Atlantic and Pacific for which they hypothesized a recent migration through the Arctic to explain the broad distribution – a hypothesis consistent with our finding representatives of this population in Churchill. Regardless, an LSR based on COI-5P data was uncertain (Figure 2b).

#### *Petalonia filiformis* (Batters) Kuntze (n = 0/7/0) (new record for Canadian Arctic and Canada) LSR: Morphology = excluded; DNA barcode = excluded

**Comment:** Interestingly, the majority of the Churchill collections (7 of 9) that are assignable to the genus *Petalonia* form a separate sister group ~3.5% divergent in COI-5P from *P. fascia* (Figure 7). Our ITS data (not shown) do not match records in GenBank for other species of this genus [31] and these specimens, in retrospect, are likely *P. filiformis* from Europe based on morphology (Figure 7). However, we would consider an LSR based on morphology as uncertain as this species was variously field-identified as *Petalonia* sp. or *Scytosiphon* sp*.* A molecular prediction for LSR awaits data from other Atlantic collections, this species being recorded only from Europe and Iceland. It may represent one of the few cases noted here for a European Atlantic component to the Churchill flora. However, in the absence of specimens from regions other than Churchill, this species is excluded from our LSR summary.

#### *Scytosiphon canaliculatus* (Setchell & N.L. Gardner) Kogame (n = 76/4/87) (new record for Canadian Pacific, Arctic & Atlantic)

**LSR: Morphology =** uncertain; **DNA barcode** = uncertain.

**Comment:***Scytosiphon* is again a genus in need of significant taxonomic revision. This species is currently reported from a variety of locations in the Pacific [32], but our records are the first reports for the Canadian Pacific, as well as for the Arctic and Atlantic Oceans. Considering its cryptic nature with other species of the genus (thus completely overlooked in the three Canadian oceans), an LSR based on morphology is uncertain (Figure 2a). This species likely accounts for many (if not all) of the records attributed to *Scytosiphon lomentaria* (Lyngbye) Link in the Arctic (e.g., [3,4,16]), although other overlooked species may also be contributing (McDevit & Saunders, unpublished data). Although COI-5P data resolved four divergent genetic groups, the Churchill collections fell into two of these, both containing collections from the Atlantic and Pacific. As such molecular data are equivocal on the LSR for the Churchill populations of this species (Figure 2b).

#### *Scytosiphon* sp._1crust (n = 0/1/0) (taxonomic work needed; possible new species or new record for Canadian Arctic)

**LSR: Morphology =** excluded; **DNA barcode** = excluded.

**Comment:** This is a unique record from Churchill and was from a brown crust that we had collected. It thus is possibly the sporophyte from a species with the typical scytosiphonacean alternation of generations [33] for which we have yet to collect the erect gametophyte stage, or an asexual species. Either way, as we have not encountered this species in the Atlantic or Pacific, it is not included in the LSR summary.

### Ectocarpales, Striariaceae

#### *Stictyosiphon soriferus* (Reinke) Rosenvinge (n = 0/1/0) (new record for Canadian Arctic)

**LSR: Morphology =** excluded; **DNA barcode** = excluded.

**Comment:** We only have a single collection of this species and it is from the Churchill region. Morphologically we can possibly ally this species to an Atlantic source [11], but we lack sufficient sampling to include it in the LSR summary.

#### *Stictyosiphon tortilis* (Ruprecht) Reinke (n = 0/9/0)

**LSR: Morphology =** excluded; **DNA barcode** = excluded.

**Comment:** This species reportedly occurs in all three oceans [16]. As we have very few collections for this species, and only from Churchill, it is not included in the LSR summary.

### Fucales, Fucaceae

#### *Fucus distichus* Linnaeus (n = 51/1/32)

**LSR: Morphology =** uncertain; **DNA barcode** = uncertain.

**Comment:** Although species of this genus are commonly reported from all the regions under consideration here [3,11,16,22], Kucera & Saunders [34] demonstrated that morphology can be very misleading in the identification of specimens for this genus in the Canadian flora. Either way, an LSR based on morphology would be uncertain (Figure 2a). With the exception of two individuals from the Atlantic, each with a single unique autapomorphy, all collections had one of three mitotypes that differed by only 0.15-0.3%. There was an Atlantic type (n = 29) and a Pacific type (n = 40), as well as a third type that was predominantly Pacific (n = 11; typically northern British Columbia), but that also contained our only barcoded specimen from Churchill and a single collection from northern Newfoundland [34]. Thus our molecular data could be interpreted as indicating a Pacific LSR for this species with migration through to the North Atlantic. However, our third mitotype matches the C2 mitotype of Coyer et al. [35], which they found in similar regions to us, but also throughout colder regions of the European North Atlantic (an area that we did not sample). Although Coyer et al. [35] predicted that this mitotype originated in the Pacific, the recent post-glacial colonization of Churchill could presumably have been from either European refugia or the Pacific region as defined here and thus an LSR based on the current molecular data is uncertain (Figure 2b).

### Laminariales, Alariaceae

#### *Alaria esculenta* (Linnaeus) Greville (n = 0/7/20)

**LSR: Morphology =** uncertain; **DNA barcode** = Pacific.

**Comment:***Alaria esculenta* is reported from all three regions under study here [3,11,16] and thus determining an LSR based on morphology is uncertain (Figure 2a). Although we have no Pacific collections for genetic analyses we infer that the Arctic collections (in this case one from the northern tip of Baffin Island, Nunavut, in addition to Churchill) have a Pacific LSR (Figure 2b). This is because there are three fixed differences in COI-5P for the Arctic collections relative to our North American Atlantic collections (n = 19), which virtually all have identical mitotypes (sampled from Maine, USA to northern Newfoundland [36]). We have also included a collection from Atlantic Europe that has a distinctive mitotype (three autapomorphies) relative to the North American collections, but which nonetheless shares the three fixed differences discussed previously with the Atlantic rather than Arctic collections, suggesting that this is not the LSR of our Churchill populations (Figure 2b). Interestingly, Lane et al. [36] recovered rubisco spacer (plastid), COI-5P (mitochondrion) and ITS (nuclear) data for a supposed collection of *A. esculenta* from Prospect Pt., Resolute Bay in the Canadian Arctic and all indications were that this plant was actually the north Pacific species *A. marginata*. Although not included in our LSR summary, this observation, if confirmed, would be for migration also of *A. marginata* from an unequivocal Pacific LSR into the Canadian Arctic.

### Laminariales, Chordaceae

#### *Chorda* sp._1*filum* (n = 0/10/0) (taxonomic work needed; possible new species or new record for Canadian Arctic)

**LSR: Morphology =** uncertain; **DNA barcode** = Pacific.

**Comment:***Chorda filum* (Linnaeus) Stackhouse is reported from all three regions under study here [3,11,16,22] and thus determining an LSR based on morphology is uncertain (Figure 2a). Interestingly, all of our “*Chorda filum*” collections from Churchill resolved as a distinct genetic group thus far unique to that region and 2.8-3.3% divergent from our Atlantic collections. It is most likely that this is a species of northern Pacific origin and accounts for records of *C. filum* in that flora [16]. Although this hypothesis remains untested, we consider the molecular data consistent with a Pacific LSR (Figure 2b) because we have not encountered this genetic group in the Atlantic despite continued barcode assessments of *C. filum* (n = 21) collections from Maine to Newfoundland.

### Laminariales, Costariaceae

#### *Agarum clathratum* Dumort. (n = 4/7/20)

**LSR: Morphology =** uncertain; **DNA barcode** = uncertain.

**Comment:** As this species is reported in the Arctic, Atlantic and Pacific Oceans [3,11,16,22], identifying an LSR based on morphology is uncertain. With the exception of a single substitution in one collection from Churchill, the COI-5P was identical in all collections rendering the likely source region equivocal by molecular analyses as well (Figure 2), but likely indicating a recent migration through the Arctic from one source region to the other.

### Laminariales, Laminariaceae

#### *Laminaria digitata* (Hudson) J.V. Lamouroux (n = 0/2/51)

**LSR: Morphology =** uncertain; **DNA barcode** = Atlantic.

**Comment:** This species has been reported from the Arctic and Atlantic regions [3,11], but not the Pacific [16,22]. However, identification on morphological grounds is problematic because this species has been clearly confused with *Saccharina groenlandica*[21], which is found in all three regions under consideration here. We thus consider an LSR on the basis of morphology uncertain (Figure 2a), while an LSR based on the barcode indicates an Atlantic origin (Figure 2b; despite sequencing eight digitate morphs of *S. groenlandica* from the Pacific region, bona fide *L. digitata* has not been encountered to date).

#### *Laminaria solidungula* J. Agardh (n = 0/9/1)

**LSR: Morphology =** excluded; **DNA barcode** = excluded.

**Comment:** One of the more morphologically distinct kelp in the Arctic, this species is reported in the Arctic, Atlantic and Pacific regions [3,11,37]; identifying an LSR based on morphology is thus uncertain. Owing to a lack of Pacific collections in our COI-5P alignment, the likely source region is also equivocal by molecular analyses although the one Atlantic collection does have an identical COI-5P to the Churchill collections. Owing to the lack of Pacific collections, and the paucity of Atlantic collections, this species is excluded from our LSR summary.

#### *Saccharina groenlandica* (Rosenvinge) C.E. Lane, Mayes, Druehl & G.W. Saunders (n = 19/11/8)

**LSR: Morphology =** uncertain; **DNA barcode** = Atlantic.

**Comment:** The geographical distribution of this species as recorded in the literature is difficult to interpret owing to considerable taxonomic confusion, but it is clear that it occurs in all three regions under consideration here [21]. Combined with its substantial phenotypic plasticity [21], the presence of this species in all three regions renders and LSR based on morphology uncertain (Figure 2a). The COI-5P data recover two groups distinguished by a single fixed difference (with the exception of a unique substitution in one Pacific isolate, GWS004436) with the Atlantic and Churchill collections distinct from our Pacific collections weakly suggesting an Atlantic LSR for the Churchill population (Figure 2b).

#### *Saccharina latissima* (Linnaeus) C.E. Lane, Mayes, Druehl & G.W. Saunders (n = 15/35/82)

**LSR: Morphology =** uncertain; **DNA barcode** = Atlantic & Pacific.

**Comment:** This species is reported in the Arctic, Atlantic and Pacific regions [3,11,16,22,37] and thus an LSR based on morphology is uncertain (Figure 2a). Molecular data have indicated that this species has established in the Churchill flora from both Atlantic and Pacific populations and is now hybridizing in the Canadian subarctic with the putative Pacific genetic signature reaching into the Canadian Atlantic [21]. An LSR based on molecular data thus has this species entering the Churchill flora from both the Atlantic and Pacific Oceans (Figure 2b).

### Sphacelariales, Sphacelariaceae

#### *Sphacelaria radicans* (Dillwyn) Harvey (n = 0/1/0)

**LSR: Morphology =** excluded; **DNA barcode** = excluded.

**Comment:** Unfortunately we have only a single barcode record for this species, which is reported for the Arctic, Atlantic and Pacific Oceans [3,11,16,22]. It is not included in our LSR summary.

#### *Sphacelaria rigidula* Kützing (n = 0/2/0) (new record for Canadian Arctic)

**LSR: Morphology =** excluded; **DNA barcode** = excluded.

**Comment:** This plant has not been recorded previously from the Canadian Arctic. As we have only two collections, and this species reportedly occurs in the Atlantic [11] and Pacific [22] regions, we lack sufficient sampling to consider its LSR in our summary.

### Tilopteridales, Halosiphonaceae

#### *Halosiphon* sp._2*tomentosus* (n = 0/1/0) (taxonomic work needed; possible new species or new record for Canadian Arctic)

**LSR: Morphology =** uncertain; **DNA barcode** = Pacific.

**Comment: *“****Halosiphon tomentosus”* (Lyngbye) Jaasund resolved as two divergent (~8%) genetic groups for the few collections we have barcoded with one mitotype confined to our Atlantic collections (n = 14) and the second unique to a Churchill collection. Until recently considered cold-water Atlantic extending into the contiguous Arctic and absent from the Pacific in distribution [11,16,22], this species was recently reported from Alaska [38]. An LSR on morphological grounds is thus uncertain (Figure 2a). Kawai & Sasaki [38] noted substantial differences in the *rbc*L sequences for Atlantic versus Pacific collections of this “species,” as also noted here for COI-5P between the Atlantic and Churchill collections. We have generated *rbc*L data for one of our Churchill collections of this morphological species (GWS005229) and it is similar to the Alaskan rather than North Atlantic isolates studied by Kawai & Sasaki [38]. We thus score the LSR as Pacific (Figure 2b).

### Tilopteridales, Tilopteraceae

#### *Haplospora globosa* Kjellman (n = 0/1/4)

**LSR: Morphology =** Atlantic; **DNA barcode** = Atlantic.

**Comment:** This species is currently reported from the Canadian Arctic and north Atlantic [3,11]. It has a relatively distinctive morphology and resolves as a single genetic group (thus far) in COI-5P analyses and, despite the few collections, can be recorded as Atlantic for its LSR based on both morphological and molecular criteria (Figure 2).

### Florideophyceae

Ahnfeltiales, Ahnfeltiaceae

#### *Ahnfeltia borealis* D. Milstein & G.W. Saunders (n = 0/7/3)

**LSR: Morphology =** uncertain; **DNA barcode** = Atlantic.

**Comment:** This recently described species was originally overlooked in the Canadian Arctic as *Ahnfeltia plicata* (discussed below), but also has attributes of the Pacific *A. fastigiata* (Endlicher) Makienko [39] and thus the LSR is considered uncertain based on morphology (Figure 2a). The molecular data are intriguing in that they suggest an Atlantic origin, but there is an interesting pattern suggesting that this may not be the case. Despite sequencing over 100 collections of *Ahnfeltia* spp. from the Atlantic region, only three were assigned to *A. borealis*. One was a drift plant on Prince Edward Island (Atlantic) and the other two were the crustose sporophytic phase encountered to the south in Massachusetts and Rhode Island, USA. Through our ongoing global macroalgal DNA barcoding project, we are finding that the sporophytic phases of red algae with heteromorphic life histories can occur sporadically in areas remote to the distributional records noted for their gametophytic counterparts, possibly being more amenable to both human-mediated transport and asexual reproduction. As such, records based on the gametophytic stages may be more meaningful when completing biogeographical comparisons along the lines of the research here, although a counter argument would certainly be true depending on the question being addressed. Thus, we suspect that this species may have a Pacific LSR. Nonetheless, we have sequenced 33 individuals for this genus from British Columbia, some looking very much like this species in field-identification, and have not had a positive hit for this genetic group and have thus scored the LSR as Atlantic pending further study (Figure 2b).

#### *Ahnfeltia plicata* (Hudson) Fries (n = 0/2/96)

**LSR: Morphology =** uncertain; **DNA barcode** = Atlantic.

**Comment:** This species is recorded from all three geographical regions under consideration here [3,11,16], although cryptic species issues will certainly impact on the accuracy of published records [39]. Given the cryptic species problems, we have scored the morphological LSR as uncertain (Figure 2a). We have sequenced 33 individuals of this genus from British Columbia, with some collections from the north at times looking very much like this species in field-identification, but which nonetheless are genetically assignable to *A. fastigiata* and not *A. plicata*. We therefore tentatively consider the LSR as Atlantic pending further study (Figure 2b).

### Corallinales, Hapalidiaceae

#### *Lithothamnion glaciale* Kjellman (n = 0/1/38)

**LSR: Morphology =** uncertain; **DNA barcode** = Atlantic.

**Comment:** Coralline crusts are among the least studied and taxonomically most confused of the algal groups that we have encountered during our global barcode surveys.

Although this particular species is reported in all three regions under consideration here [3,11,16], to score an LSR based on morphology as anything other than uncertain is naïve (Figure 2a). We have sequenced numerous coralline crusts from northern British Columbia (n = 52 from Haida Gwaii, BC, alone) and have not had a positive hit for this genetic group and consider the LSR as Atlantic pending further study (Figure 2b).

#### *Phymatolithon lenormandii* (Areschoug) W.H. Adey (n = 0/3/60)

**LSR: Morphology =** uncertain; **DNA barcode** = Atlantic.

**Comment:** All of our comments above for *L. glaciale* apply equally to this species; the morphological and molecular LSR’s are listed as uncertain and Atlantic, respectively (Figure 2).

### Acrochaetiales, Acrochaetiaceae

#### *Acrochaetium* sp. (n = 0/1/0) (taxonomic work needed; possible new species or new record for Canadian Arctic)

**LSR: Morphology =** excluded; **DNA barcode** = excluded.

**Comment:** The genus *Acrochaetium*, as is true of most of the (typically) microscopic reds, requires considerable taxonomic study. It is possible that this relatively common species (despite having only one barcoded collection, we have observed it epiphytically on many of our other collections during identification) accounts for some records of *A. microscopicum* from the Canadian Arctic [3], but in some respects our collections better match the Pacific *A. hirsutum* as detailed in Garbary et al. [40] (Clayden & Saunders, unpublished data). This single collection from Churchill is thus of uncertain origin on both morphological and, owing to a paucity of data, molecular grounds and is not included in the LSR summary.

### Palmariales, Palmariaceae

#### *Devaleraea ramentacea* (Linnaeus) Guiry (n = 0/3/23)

**LSR: Morphology =** excluded; **DNA barcode** = excluded.

**Comment:** Occurring in all three geographical regions under study [3,11,16], the source region for this species based on morphology is uncertain. Although our three collections from Churchill have a unique mitotype, it falls among other mitotypes from the Atlantic. In the absence of any collections attributable to this morpho-species from the Pacific region we have excluded this species from our LSR summary.

#### *Palmaria palmata* (Linnaeus) Kuntze (n = 0/7/28)

**LSR: Morphology =** uncertain; **DNA barcode** = Atlantic.

**Comment:***Palmaria palmata* is widely recorded in the Atlantic and Arctic regions under study here [3,11]. Reports of this species from the Pacific are now largely attributed to misidentification with Pacific species of this genus [41], but as many of these species are morphologically very similar it was cautioned that additional study was needed to determine whether or not bona fide *P. palmata* is present in the more northerly reaches of the Pacific region. As such, an LSR would not be easily determined on the basis of morphology alone (Figure 2a). Although there was a fair degree of variability for COI-5P within this species, 0 – 1.4%, most of that divergence was between European and North American Atlantic/Arctic collections, the latter with only 0 – 0.62% divergence. The seven Churchill collections were identical and clustered with the most common Atlantic Canada mitotype. Further, barcodes generated from 75 isolates of *Palmaria* from the Pacific region were distinct from *P. palmata*. Thus the genetics point to an Atlantic (North American) LSR for the Churchill collections (Figure 2b).

### Palmariales, Rhodophysemataceae

#### *Rhodophysema kjellmanii* G.W. Saunders & Clayden (n = 0/2/0)

**LSR: Morphology =** excluded; **DNA barcode** = excluded.

**Comment:** Although reported from all three regions under discussion here [3,11,16], we have only managed to collect this species in Churchill. Being present in both the Atlantic and Pacific regions, morphology is uncertain with regards to the LSR. No conclusions can be framed from the COI-5P data owing to the paucity of samples and this species is excluded from the LSR summary.

### Ceramiales, Ceramiaceae

#### *Scagelia sp.* (n = 25/18/26) (taxonomic work needed; possible new species or new record for Canadian Arctic)

**LSR: Morphology =** uncertain; **DNA barcode** = Pacific.

**Comment:** Past records attributed to *Scagelia pylaisaei* (Montagne) M.J. Wynne in the Atlantic [11] and Arctic [3,16] on the basis of morphology should be considered with caution. Indeed, researchers cannot even agree on how many species of *Scagelia* to recognize in Canada and their respective distributions in our three oceans (e.g., compare [11,22,42] to list only a few of the papers embroiled in this taxonomic conundrum). Determining an LSR based on morphology can thus be ruled out (Figure 2a). Bruce & Saunders [43] have discovered that none of the current perspectives in the literature are correct and, at least for the purposes of the current report, that Churchill plants are Pacific in their origin based on both COI-5P and ITS sequence data rendering this the LSR (Figure 2b).

### Ceramiales, Delesseriaceae

***Phycodrys fimbriata *(Kuntze) Kylin (n = 0/8/35) (new record for Canadian Arctic) LSR: Morphology =** uncertain; **DNA barcode** = Atlantic.

**Figure 8:**
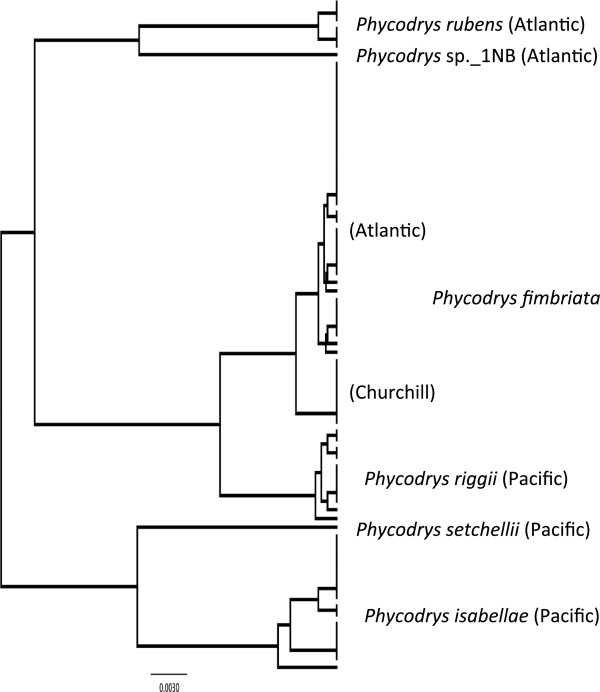
**Overlooked diversity and the biogeographical distribution of the associated mitotypes for specimens assigned to morpho-species of the genus *Phycodrys*.
**

**Comment:** As with most of the taxa discussed here, this genus is in need of a thorough taxonomic evaluation. We have barcoded 81 collections from North America and include data from *Phycodrys fimbriata* (n = 43), *P. isabellae* R.E. Norris & M.J. Wynne (n = 18), *P. riggii* Gardner (n = 11)*, P. rubens* (Linnaeus) Batters (n = 6), *P. setchellii* Skottsberg (n = 1) and an unidentified species from New Brunswick (sp._1NB; n = 2) in our molecular analysis owing to the morphological uncertainty surrounding these taxa (Figure 8). Identifying an LSR on morphology would unequivocally be uncertain (Figure 2a). Currently, only *P. rubens* is recognized in Atlantic Canada [11], but we resolved three species in these waters (Figure 8); *P. rubens* actually being relatively rare compared to *P. fimbriata* (we apply this name to this genetic group based on personal communications with M. Hommersand) a result consistent with an earlier study (van Oppen et al. [44]; allowing for a correct rooting between their *P. riggii* and *P. rubens* clusters rather than within the former as depicted in their Figure 3). In the Canadian Arctic, only *P. rubens* was recorded [3], but we did not encounter this species in Churchill and those records are possibly attributable to *P. fimbriata*. Lindstrom [16] recognized both *P. riggii* and *P. rubens* in the Arctic, Atlantic and Pacific Oceans, but the former is now regarded as a taxonomic synonym of *P. fimbriata*[32]. Although, *P. fimbriata* and *P. riggii* share similar gross morphology, they formed discrete sister genetic groups in our COI-5P trees (Figure 8) (only 1.4-2.2% divergent; additional markers need to be tested prior to deciding on species distinction for these two groups). It is noteworthy that the Churchill population has a unique mitotype sister to the Atlantic mitotypes (Figure 8), which opens the possibility that *P. fimbriata* migrated into the Canadian Arctic from either Europe or the northerly-most reaches of the Pacific region (poorly sampled here) or from a northern glacial refugium. Testing these hypotheses will require samples from additional populations; for now the molecular data are most consistent with an Atlantic LSR for Churchill *P. fimbriata* (Figure 2b).

### Ceramiales, Rhodomelaceae

#### *Odonthalia dentata* (Linnaeus) Lyngbye (n = 0/7/4)

**LSR: Morphology =** excluded; **DNA barcode** = excluded.

**Comment:** Another genus in need of taxonomic evaluation, especially in the Pacific region (our various genetic groups contradict morpho-species in the local guide of Gabrielson et al. [22] consistent with their assertion that work is needed on these species in that area), there is only one species reported in the Canadian Arctic [3] and Atlantic [11], *O. dentata,* which was consistent with our genetic data. Unfortunately, this morpho-species is also reported widely in the north Pacific region [16] such that an LSR cannot be determined by morphology alone. Given its distribution only into the cold waters of the Atlantic Provinces, the Pacific region does represent a likely source region, but without collections from that region, we have excluded this species from our LSR summary.

#### *Polysiphonia arctica* J. Agardh (n = 0/7/2)

**LSR: Morphology =** excluded; **DNA barcode** = excluded.

**Comment:** This cold-water (considered an Arctic endemic) species is reported in all of the regions under study here [3,11,16] and we have samples from the Atlantic (both from Newfoundland) and Churchill (including one from the northern tip of Baffin Island, Nunavut). Considering the lack of samples for this relatively distinct morpho-species from the Pacific, we have excluded this species from our LSR summary.

#### *Polysiphonia stricta* (Dillwyn) Greville (n = 1/6/54) (taxonomic work needed; possible new species or new records for Canadian Arctic)

*Polysiphonia* sp._*1stricta*

**LSR: Morphology =** uncertain; **DNA barcode** = uncertain.

*Polysiphonia* sp._*3stricta*

**LSR: Morphology =** uncertain; DNA barcode = Atlantic.

**Comment:** This morpho-species has been reported in all three regions under study here [3,11,16,22], although with some uncertainty for British Columbia where we have had only a single positive match (*Polysiphonia* sp._1*stricta*; despite generating barcode data for 141 collections for this genus from this region). Savoie & Saunders [45] have uncovered three genetic groups under this species name, and thus morphology is not useful for inferring the LSR for populations of either of the cryptic species found in Churchill (sp._1 n = 4; sp._3 n = 2) (Figure 2a). *Polysiphonia* sp._3*stricta* had only Arctic and Atlantic collections suggesting the latter as LSR while *Polysiphonia* sp._1*stricta* was collected from all three regions leaving an LSR based on molecular data uncertain (Figure 2b).

#### *Rhodomela confervoides* (Hudson) P.C. Silva (n = 0/1/57)

**LSR: Morphology =** uncertain; **DNA barcode** = Atlantic.

**Figure 9:**
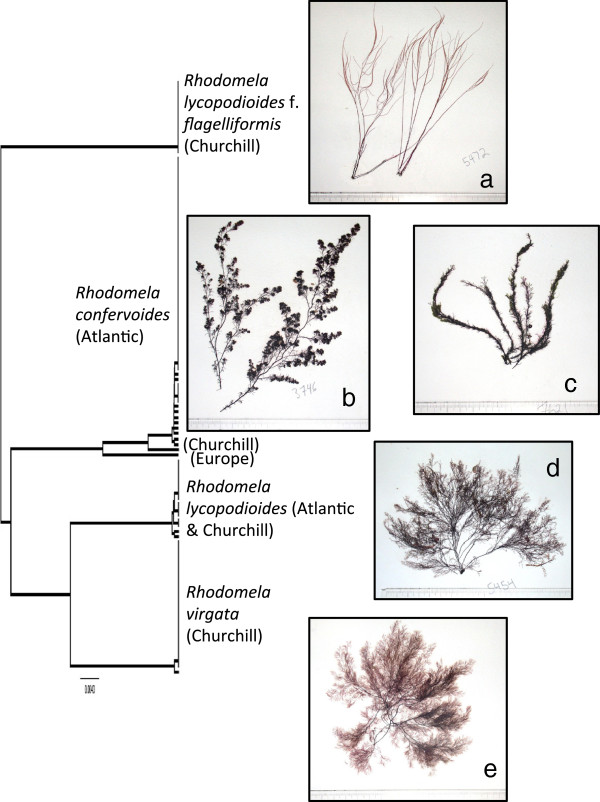
**Overlooked diversity and the biogeographical distribution of the associated mitotypes for specimens assigned to morpho-species of the genus *Rhodomela*.** **a-e**. Representative morphologies for *Rhodomela* spp. from Canada. **a**. Specimen of *Rhodomela lycopodioides* f. *flagelliformis* from Churchill (GWS005472). **b**. Typical winter morph of *Rhodomela confervoides* (GWS003746). **c**. Specimen from north Atlantic waters field identified as *R. lycopodioides*, but genetically assigned to *R. confervoides* (GWS007621). **d**. Specimen of *Rhodomela lycopodioides* from Churchill (GWS005454). **e**. Specimen of *Rhodomela virgata* from Churchill (GWS005455). Scale = centimeter ruler.

**Comment:** In its typical form *R. confervoides* (Figure 9b) is distinct and reported in the Canadian Arctic [3] and Atlantic [11], but not Pacific region [16,22]. However, all of our plants field identified as *R. lycopodioides* (sensu Maggs & Hommersand [46], p. 296), a second species of this genus but reported from all three regions under consideration here [3,11,16], from Newfoundland extending south were simply *‘lycopodioides’*-morphs of *R. confervoides* (Figure 9c). In contrast, our Arctic specimens assigned to *R. lycopodioides* in the field resolved in three distinct genetic groups (discussed below) none of which were particularly similar to the specimen from near the type locality noted in Maggs & Hommersand ([46]; these authors discuss the uncertain status of *R. lycopodioides*) (Figure 9). An LSR based on morphology is thus uncertain (Figure 2a) while an Atlantic LSR is most likely based on the molecular data currently in hand (Figure 2b). One of our Atlantic collections for *R. confervoides* is from Europe and resolves as sister to our Arctic/Atlantic mitotypes (Figure 9) indicating that the Churchill plant has likely originated from the North American side of the Atlantic. The single Churchill mitotype was nonetheless sister to the Atlantic collections leaving open the possibility of a migration from the northern-most reaches of the Pacific region (not reported for that area, but poorly sampled) or from a northern glacial refugium; hypotheses that can only be tested with additional collections from all regions.

#### *Rhodomela lycopodioides* (Linnaeus) C. Agardh complex (taxonomic work needed; possible new species or new records for Canadian Arctic)

This ‘morpho-species’ resolved as three distinct genetic groups (Figure 9). Being reported from all three regions under study here [3,11,16,22] an LSR based on morphology would be uncertain for all species. Data acquired from the published literature (especially checklists lacking images and/or vouchers) would be difficult to interpret for this complex as it would be uncertain which species of these three was actually being observed (not to mention the *‘lycopodioides’-*morphs of *R. confervoides* discussed above; Figure 9c). Further, any taxonomic revision of the morpho-species *R. lycopodioides* must include collections from the type locality (and morphology), as well as consider Russian records currently assigned to *R. sibirica* A.D. Zinova & Vinogradova and *R. tenuissima* (Ruprecht) Kjellman, which both have superficial morphological resemblance to the various genetic groups here [47]. We have DNA barcoded 36 collections from British Columbia tentatively assigned to the last mentioned species [22], however, these sequences group with species of *Neorhodomela* rather than *Rhodomela* in preliminary trees and the actual plants are not a good morphological match to the detailed description for *R. tenuissima* in Masuda [48]. Clearly considerable taxonomic work is required in this complex. Given the lack of samples from the Pacific region for this complex in the current study, all three are excluded from our LSR summary analysis.

#### *Rhodomela lycopodioides* (Linnaeus) C. Agardh *sensu stricto* (n = 0/11/4)

**LSR: Morphology =** excluded; **DNA barcode** = excluded.

**Comment:** One of three genetic groups that was loosely field identified as *R. lycopodioides* (Figure 9), this species matches the general vegetative attributes outlined by Kjellman [1], notably the adventitious sickle-shaped branches on older axes, for *R. lycopodioides* f. *typica* (Figure 9d). We thus assign this genetic group to this species until detailed taxonomic review of this genus is completed. Most notably, it needs to be established whether or not Kjellman’s view of this species matches the type specimen from Scotland [46].

#### *Rhodomela lycopodioides* f. *flagellaris* Kjellman (n = 0/14/0)

**LSR: Morphology =** excluded; **DNA barcode** = excluded.

**Comment:** The second of three genetic groups that was loosely field-identified as *R. lycopodioides* (Figure 9), this species matches the general vegetative attributes outlined by Kjellman [1] and reiterated by Lund [49] for *R. lycopodioides* f. *flagellaris* (Figure 9a). Despite the presence of adventitious sickle-shaped branches on older axes, which Kjellman considered a diagnostic trait of *R. lycopodioides* f. *typica*, this species is morphologically and genetically distinct (Figure 9) and will need to be assigned species status following a detailed taxonomic review of this genus. This is the only species of *Rhodomela* that we encountered during a trip to the high Arctic (northern tip of Baffin Island, Nunavut).

#### *Rhodomela virgata* Kjellman (n = 0/27/0)

**LSR: Morphology =** excluded; **DNA barcode** = excluded.

**Comment:** The last of three genetic groups that was loosely field identified as *R. lycopodioides* (Figure 9), this species matches the general vegetative attributes outlined by Kjellman [1] for *R. virgata* (Figure 9e), and matches the particular size (~80-90 μm in length) and distribution (along the “sides of the long shoots”) of the tetrasporangia as detailed in Rosenvinge [50]. We thus assign this genetic group to this species until detailed taxonomic review of this genus is completed.

### Ceramiales, Wrangeliaceae

*Ptilota gunneri* P.C. Silva, Maggs & L.M. Irvine (n = 0/1/2) (new record for Canadian Arctic)

**LSR: Morphology =** uncertain; **DNA barcode** = Atlantic.

**Figure 10:**
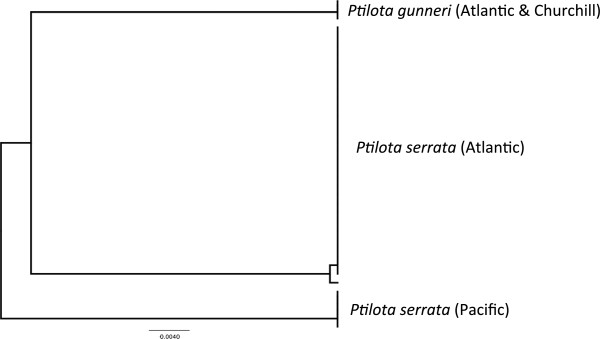
**Overlooked diversity and the biogeographical distribution of the associated mitotypes for specimens assigned to morpho-species of the genus *Ptilota*.
**

**Comment:** Again taxonomic confusion has masked the presence of this species in the Canadian Arctic (and Atlantic; [51]). *Ptilota serrata* is reported from the Arctic, Atlantic and Pacific Oceans [3,11,16,22], being the only species of this genus reported from the Canadian Arctic and Atlantic, and we generated 51 COI-5P sequences for specimens assigned to this morpho-species from the Arctic (n = 1), Atlantic (n = 45) and Pacific (n = 5) regions. However, COI-5P resolved three distinct genetic groups that are divergent from one another by 6-10% and thus unequivocally distinct species (Figure 10). It is clear that Pacific (thus far only British Columbia included in our analyses) plants included in *P. serrata* sensu lato need to be placed in a separate species, while Atlantic populations are assignable to *P. serrata sensu stricto* (n = 43) and the European species *P. gunneri* (n = 2) with the single Churchill collection joining the latter. Morphology has thus clearly not worked and cannot be used to determine an LSR (Figure 2a), while the molecular data are consistent with an Atlantic LSR (Figure 2b). Further, our Churchill and Canadian Atlantic collections have identical COI-5P mitotypes to collections from Europe, which possibly indicates that this species has a European Atlantic origin. Finally, it is possible that bona fide *P. serrata* occurs in the Canadian Arctic and perhaps even the northern reaches of the Pacific region under study here, but we have not collected it in these regions to date.

### Gigartinales, Dumontiaceae

#### *Dilsea socialis* (Postels & Ruprecht) Perestenko (n = 3/12/10)

**LSR: Morphology =** uncertain; **DNA barcode** = uncertain.

**Comment:** This species was recorded by Taylor ([2] as *D. integra* (Kjellman) Rosenvinge) (and presumably Lee [9]) as essentially an Arctic species (two of our twelve collections from that region being from the northern tip of Baffin Island, Nunavut), which he defined as a species distributed from northern Newfoundland northward (we have collections from St. Margarets Bay, Nova Scotia, but no further south). However, published molecular analyses [52,53] indicate that *D. integra* is conspecific with *D. socialis*, a species distributed with certainty in the northern-most Pacific region from Kamchatka, Russia through the Bering Sea with barcoded records from the Beaufort Sea, Alaska included in our analyses (n = 3) [20,53]. Identifying an LSR on morphology is thus not possible (Figure 2a). Unfortunately, the COI-5P sequences are virtually identical in all of the specimens included here. The near homogeneity of mitotypes throughout this species’ range is consistent with a recent migration between the Atlantic and Pacific regions from a single LSR, but the direction of any such migration cannot be ascertained with the data in hand. Current molecular data are thus uncertain with regards to the LSR for this species (Figure 2b).

### Gigartinales, Phyllophoraceae

#### *Coccotylus brodiei* (Turner) Kützing (n = 0/1/40)

**LSR: Morphology =** excluded; **DNA barcode** = excluded.

**Comment:** Prior to the DNA barcode study of Le Gall & Saunders [54], *C. brodiei* was considered a taxonomic synonym of *C. truncatus* (Pallas) M.J. Wynne & Heine (e.g., [11]), a species recorded in all three geographical regions under consideration here [3,11,16]. Further, these two species overlap in their morphology and thus are difficult to identify in the field. Identification of an LSR based on morphology is thus uncertain. The loan collection from Churchill resolves deeply among collections with the most common Atlantic mitotype, which is consistent with this species having an Atlantic LSR, based on molecular data. However, we lack collections of this morpho-species from the Pacific region and thus exclude it from our LSR summary.

#### *Coccotylus truncatus* (Pallas) M.J. Wynne & Heine (n = 0/10/13)

**LSR: Morphology =** excluded; **DNA barcode** = excluded.

**Comment:** From a morphological perspective, the comments under *C. brodiei* apply equally here with the LSR being uncertain. The collections from the Arctic region in this case additionally include one plant from the northern tip of Baffin Island, Nunavut (this apparently being the ‘more Arctic’ of the two *Coccotylus* spp.). There is an interesting diversity of mitotypes in the northern collections with some plants forming a distinct Arctic mitotype (only one fixed difference, but possibly signature of a Pacific mitotype?), but which is nonetheless nested among other Atlantic mitotypes consistent with the LSR being Atlantic. Again, because we lack collections of this morpho-species from the Pacific region we exclude it from our LSR summary.

## Discussion

Our initial objective was strictly to use DNA barcode data to acquire a better understanding of seaweed diversity in Churchill. In this regard, we were largely successful and recorded 57 genetic groups where only ~50 morpho-species were identified in the field (with barcode data not generated for six of the latter). The discrepancy in these values was attributable to phenotypic plasticity, overlooked diversity, range extensions for some species and recognition that some genera are in need of substantial taxonomic revision in the North American flora. Whereas we were able to resolve some of these taxonomic issues and apply names to records from Churchill (e.g., *Chordaria chordaeformis*), we have generated far more taxonomic problems than we have resolved. These will serve as the foundation for a number of future studies dealing specifically with taxonomic issues for the various species and genera found in Canadian waters. On the positive, we have also recorded eight new distributional records for the Churchill region, as well as some for the Canadian Atlantic and Pacific regions through our efforts, while 17 genetic species/complexes that are from a variety of overlooked species groups will most certainly increase these values following detailed taxonomic study.

Perhaps more interesting to the general community were the inferences that we were able to make on the Likely Source Regions for the Churchill populations of the various species that have re-colonized this area following the last glacial retreat.

We must start by acknowledging that this aspect of the current manuscript has several weaknesses. Foremost, we have genetic data for a limited number of samples, which is exacerbated by a strategy to get as many species as we could (DNA barcode objective) at the expense of replication within species (necessary for biogeographical studies). A particular aspect in need of improvement would be the addition of samples for the pertinent species (Additional file 1) from the European Atlantic and the northern most reaches of the ‘Pacific region’ as defined here (e.g., Bering Sea, Chukchi Sea, etc.). Second, our goal was strictly to identify the Likely Source Region for species present in Churchill, all of which would have had to migrate to that region following the last glacial retreat (~10,000 years ago [6]). As such, longer-term phylogeographic patterns of Pacific contributions to the Canadian Arctic and Atlantic floras were not considered. For example, whereas the current population of *P. washingtoniensis* in Churchill has mitotypes consistent with contemporary Atlantic rather than Pacific populations (hence an Atlantic LSR in our summary), it is parsimonious (based on the ‘phylogeny’ in Figure 4) to conclude that this species had previously migrated from the Pacific into the Atlantic during an earlier warming event. Finally, the genetic markers that we used, although widely established as species-level barcode markers in the groups under study here and thus suitable for a floristic survey of a region [23], are not necessarily optimal markers for population level studies such that resolution was surely lost.

Nonetheless, we feel that our efforts have merit. First, for most of the genetic species considered here (Additional file 1) the issue of an LSR into the Churchill region was a species-level rather than population level question. In many instances these involved overlooked, but nonetheless distinct, genetic species that were masked under a single morpho-species (e.g., *Desmarestia aculeata* complex, Figure 3) with the molecules providing a clear indication of an LSR. Although more sampling from all regions is necessary to ensure that the novel species are not more widespread than currently interpreted based on the collections available, this study does set a foundation for future research on this question going forward. Second, in some cases we had fairly substantial number of samples coupled with reasonable within genetic group COI-5P variation such that population inferences were not unreasonable (e.g., *Pylaiella washingtoniensis*; Figure 4). Allowing for these caveats, we can note a number of significant trends in our data.

Based on our data we hypothesized that ~21% of the flora in the Canadian Arctic has recolonized through recent migrations from the Pacific region. This finding agrees with the predictions of the Adey et al. [12] Thermogeographic Model and the notion of trans-Arctic migrations having a strong Pacific to Atlantic migrational bias. This result also lessens the biogeographical ‘paradox’ regarding the Canadian Arctic benthic flora relative to fauna discussed in the literature [12], also see [55], which was also challenged by Lindstrom [16]. In fact, it is highly likely that our current value represents an underestimate as a number of key species had to be excluded from our analyses owing to a lack of collections from the most northerly Pacific region. Many of the so-called Arctic species [2] are now considered iconic examples of Pacific contributors to the Arctic and Atlantic floras [12]. If we include even a few of these, such as the brown alga *Laminaria solidungula* and the red algae *Devaleraea ramentacea*, *Odonthalia dentata*, *Polysiphonia arctica* and *Coccotylus truncatus* as being of putative Pacific origin, then the percent contribution from that source region climbs to 33%. *Dilsea socialis*, which was recorded as an uncertain LSR based on the molecular data at hand, and *Turnerella pennyi*, for which we were unable to generate barcode data here, were also noted as exemplar species in Adey et al. [12] and if treated as Pacific LSR’s would raise the percentage contribution from that region to 37%. Interestingly, the seven previous species are largely distributed only in the more northerly reaches of the Pacific region as defined here (e.g., Bering Sea), as are three of the six species for which we have argued a clear Pacific LSR (viz., *Alaria esculenta*, *Chorda* sp._1*filum*, *Halosiphon* sp._2*tomentosus*), and are not associated with the British Columbia flora. This biogeographical pattern is consistent with results from a DNA barcode survey of marine polychaetes suggesting a clear distinction between the biota in British Columbia relative to the northern reaches of the Pacific region and the Canadian Arctic [18]. We additionally uncovered a number of taxa for which the molecular LSR was recorded as ‘uncertain’ (Figure 2b), including *Agarum clathratum*, *Petalonia fascia, Punctaria* sp._2GWS and *Scytosiphon canaliculatus*, that had virtually identical within species mitotypes for representative collections from all regions under study here suggesting recent migrations for these species between the Atlantic and Pacific through the Arctic although the direction remains uncertain (also true for *Dilsea socialis*, which was considered previously). If even half of these species had a Pacific LSR, the percentage contribution to the Canadian Arctic flora from that region would rise to 38.5%, while if all four derived from that region a Pacific region contribution of 44% would be realized. These last-mentioned species point to the pivotal stage of a trans-Arctic migration, i.e., evidence for the Pacific flora reaching into the Atlantic region as documented for *Saccharina latissima*[21]. Inevitably some of these species may have had their mitotype distributions impacted by human-mediated introductions (e.g., *Ulva* spp. [28]), which will have to be considered as these data and analyses continue to develop. Additionally, only with enhanced sampling from the Pacific region, especially the northern most areas that are the least sampled among those under discussion here [16], will these hypotheses be further tested and a more complete picture of the recent source populations for the Canadian Arctic flora realized.

Another flora in need of intense sampling relative to the Canadian Arctic flora is that of the European Atlantic. We only had limited comparative COI-5P data from Europe for a few representatives of *Alaria marginata, Palmaria palmata, Saccharina latissima* and *Rhodomela confervoides.* However, in all four cases the COI-5P data collected to date indicated that the European specimens were distinct from the North American Atlantic and Churchill populations. This indicates that for these species the Churchill region was repopulated from the ‘relict’ North American Atlantic flora and not the European flora. In contrast, barcode data for *Ptilota gunneri* are consistent with a European source for both the Canadian Atlantic and Arctic populations. Further, our putative discovery of the European species *Petalonia filiformis* in Churchill, but not the Canadian Atlantic, is consistent with a European source for that species into our Arctic waters. Again, such hypotheses await the generation of comparative data for European conspecifics of all of the species identified in our Churchill survey. We certainly look forward to these new data and further exploration of the nascent hypotheses that we have framed here.

## Conclusion

In summary, we have uncovered a number of new records for the Churchill region, as well as a number of taxa in need of detailed taxonomic study before an accurate inventory of that flora can ultimately be prepared. The beguiling morphological chaos that we have uncovered in attempting to complete this task should serve as a cautionary note to both systematists and ecologists interested in this depauperate, but infinitely interesting seaweed flora. We expect that the previous warning is not remotely unique to this algal flora, as is becoming obvious through ongoing global macroalgal DNA barcode surveys (Saunders unpublished observations). Further, our recognition of a strong Pacific component in the Churchill flora in contrast to previous floristic surveys and guides [2,3,11] is consistent with predictions from the Thermogeographic Model being advocated by Adey and colleagues to explain the phylogeography, biogeography and biodiversity of macroalgal species in the Canadian Arctic and Atlantic [5,12], as well as longer standing orthodoxy for marine invertebrates and fish [5,12-14]. This result has significance not only for understanding the current biodiversity and biogeography of seaweed species in these waters, but also ramifications for the inevitable future changes to both of these floras in light of climate change [14].

## Competing interests

The authors declare that they have no competing interests.

## Authors’ contributions

Both authors were key participants in the two Churchill collecting trips. DCM generated most of the molecular data in this study and reviewed drafts of this manuscript. GWS conceived the project and completed the majority of the manuscript preparation, data analyses and the alpha taxonomic assessment of the collections. Both authors have read and approved the final draft of the manuscript.

## Authors’ information

GWS is a Professor and Research Chair in the Department of Biology at the University of New Brunswick (UNB); he is interested in varied aspects of macroalgal systematics, biodiversity and biogeography. DCM completed his PhD studies under GWS’s supervision at UNB working on the DNA barcoding of brown macroalgae in Canada and is currently a Professor at Edison State College in Fort Myers, Florida; he is interested in marine biodiversity.

## Supplementary Material

Additional file 1 **Specimen list.** Collections from Churchill for which DNA barcodes were generated. Ulvophyceae used the markers *tufA* and *rbc*L-3P as indicated, Phaeophyceae and Rhodophyta used COI-5P. Click here for file
