# Supplementary material for: DNA barcoding unmasks overlooked diversity improving knowledge on the composition and origins of the Churchill algal flora
Source: BMC Ecol. 2013 Mar 16;13:9. doi: 10.1186/1472-6785-13-9 (PMC3606624; doi:10.1186/1472-6785-13-9)
Supplement: Additional file 1 — Specimen list. Collections from Churchill for which DNA barcodes were generated. Ulvophyceae used the markers tufA and rbcL-3P as indicated, Phaeophyceae and Rhodophyta used COI-5P. [file 1472-6785-13-9-S1.docx]

Additional file 1. Collections from Churchill for which DNA barcodes were generated. Ulvophyceae used the markers *tufA* and *rbc*L-3P as indicated, Phaeophyceae and Rhodophyta used COI-5P.

| BOLD | Species | Habitat | GenBank |
| --- | --- | --- | --- |
| Ulvophyceae |  |  |  |
| Ulotrichales |  |  |  |
| Ulotrichaceae |  |  |  |
| ULVA159-09 | *Acrosiphonia* sp._3GWS | Subtidal (6 m) on rock | JX572165^1^ |
| ULVA171-09 |  | Mid intertidal on cobble in freshwater runoff | JX572164^1^ |
| ULVA191-09 | *Acrosiphonia* sp._6GWS | Mid intertidal on cobble in freshwater runoff | JX572166^2^  JX572168^2^ |
| ULVA182-09 | *Spongomorpha aeruginosa* (Linnaeus) C. Hoek | Subtidal (3 m) on rock | HQ610277^2^  HQ603512^2^ |
| ULVA203-09 | *Ulothrix flacca* (Dillwyn) Thuret | Mid intertidal pools on rock | JX572169^3^ |
| Ulvales |  |  |  |
| Kornmanniaceae |  |  |  |
| ULVA515-09 | *Blidingia* sp._5GWS | Intertidal on rock in area of freshwater runoff | HQ610241^1^ |
| Ulvaceae |  |  |  |
| ULVA099-09 | *Ulva lactuca* Linnaeus | Subtidal (5 m) on rock | HQ610328^2^  HQ603564^2^ |
| ULVA517-09 |  | Subtidal (2 m) on rock | HQ610347^2^  HQ603581^2^ |
| ULVA147-09 |  | Subtidal (6 m) on rock | HQ610327^2^  HQ603563^2^ |
| ULVA227-09 |  | Drift | HQ610359^2^  HQ603593^2^ |
| ULVA516-09 | *Ulva prolifera* O.F. Müller | Low upper intertidal on rock | HQ610397^2^  HQ603633^2^ |
| ULVA123-09 |  | Upper intertidal fringe carpet on brackish basin | HQ610401^2^  HQ603637^2^ |
| ULVA519-09 |  | Mid intertidal pools on pebble | HQ610396^2^  HQ603632^2^ |
| ULVA215-09 |  | High intertidal pools on pebble | JX572167^3^ |
| ULVA520-09 |  | Upper intertidal on cobble | HQ610395^2^  HQ603631^2^ |
| Phaeophyceae |  |  |  |
| Desmarestiales |  |  |  |
| Desmarestiaceae |  |  |  |
| MACRO957-08 | *Desmarestia* sp._*1aculeata* (Linnaeus) J.V. Lamouroux | Drift | JX571964 |
| MACRO269-06 |  | Drift | JX572144 |
| MACRO273-06 |  | Subtidal (6 m) on rock | JX572117 |
| MACRO274-06 |  | Subtidal (6 m) on rock | JX572000 |
| MACRO348-06 |  | Subtidal (6 m) on rock | JX572088 |
| MACRO960-08 |  | Subtidal (6 m) on rock | JX571965 |
| MACRO276-06 |  | Subtidal (6 m) on rock | JX572045 |
| MACRO279-06 |  | Subtidal (13 m) on rock | JX571990 |
| MACRO280-06 |  | Subtidal (13 m) on rock | JX572065 |
| MACRO962-08 |  | Subtidal (13 m) on rock | JX572080 |
| MACRO1045-08 |  | Drift | JX571963 |
| MACRO1062-08 |  | Drift | JX572155 |
| Ectocarpales |  |  |  |
| Acinetosporaceae |  |  |  |
| MACRO397-06 | *Pylaiella* sp._*1littoralis* (Linnaeus) Kjellman | Subtidal (1 m) on *Fucus* | JX572158 |
| MACRO399-06 |  | Subtidal (1 m) on *Fucus* | JX572035 |
| MACRO400-06 |  | Drift | JX572077 |
| MACRO401-06 |  | Subtidal (1 m) on *Fucus* | JX572139 |
| MACRO347-06 |  | Mid lower pools on *Fucus* | JX572021 |
| MACRO393-06 | *Pylaiella washingtoniensis* Jao | Subtidal (3 m) on *Fucus* | JX572029 |
| MACRO345-06 |  | Subtidal (3 m) on *Fucus* | JX572091 |
| MACRO350-06 |  | Subtidal (6 m) on *Ahnfeltia* | JX572006 |
| MACRO351-06 |  | Subtidal (6 m) on *Ahnfeltia* | JX572111 |
| MACRO353-06 |  | Subtidal (6 m) on rock | JX572102 |
| MACRO354-06 |  | Subtidal (13 m) on cobble | JX572124 |
| MACRO1152-09 |  | Subtidal (18 m) on mussels | HM890861 |
| MACRO682-07 |  | Subtidal (18 m) on mussels | JX572042 |
| MACRO1153-09 |  | Mid intertidal on cobble in freshwater runoff | HM890862 |
| MACRO1154-09 |  | Mid intertidal on cobble in freshwater runoff | HM890863 |
| MACRO1028-08 |  | Mid intertidal on cobble in freshwater runoff | JX572064 |
| MACRO1029-08 |  | Mid intertidal pools on rock | JX571962 |
| MACRO696-07 |  | Drift | JX572151 |
| MACRO702-07 |  | Lower intertidal pools on rock | JX571989 |
| MACRO1438-09 |  | Subtidal (6 m) on drift moss | HM891066 |
| MACRO1054-08 |  | Subtidal (5 m) on rock | JX572022 |
| Chordariaceae |  |  |  |
| MACRO611-07 | *Chordaria chordaeformis* (Kjellman) Kawai & S.H. Kim | Subtidal (6 m) on rock | JX572107 |
| MACRO254-06 |  | Subtidal (6 m) on rock | JX572015 |
| MACRO258-06 |  | Subtidal (1 m) on rock | JX571979 |
| MACRO349-06 |  | Subtidal (6 m) on rock | JX572087 |
| MACRO278-06 |  | Subtidal (6 m) on rock | JX572097 |
| MACRO284-06 |  | Subtidal (6 m) on rock | JX572104 |
| MACRO1401-09 |  | Mid intertidal on cobble in freshwater runoff | HM891037 |
| MACRO683-07 |  | Mid intertidal on cobble in freshwater runoff | JX572130 |
| MACRO1379-09 |  | Subtidal (6 m) on rock | HM891021 |
| MACRO688-07 |  | Subtidal (6 m) on mussel | JX572110 |
| MACRO689-07 |  | Subtidal (6 m) on rock | JX571998 |
| MACRO1447-09 |  | Mid intertidal pool on rock | HM891073 |
| MACRO1047-08 |  | Low intertidal pool on rock | JX572098 |
| MACRO1049-08 |  | Low intertidal pool on rock | JX571994 |
| MACRO1051-08 |  | Lower intertidal pools on rock | JX572007 |
| MACRO1052-08 |  | Lower intertidal pools on rock | JX572003 |
| MACRO1053-08 |  | Subtidal (5 m) on rock | JX571970 |
| MACRO1449-09 |  | Mid pool on rock | HM891075 |
| MACRO1059-08 |  | Mid pool on rock | JX572129 |
| MACRO1060-08 |  | Mid pool on rock | JX572048 |
| MACRO956-08 | *Chordaria flagelliformis* (O.F. Müller) C. Agardh | Subtidal (5 m) on rock | JX572112 |
| MACRO263-06 |  | Subtidal (1 m) on limpet | JX572052 |
| MACRO398-06 |  | Subtidal (1 m) on rock | JX572023 |
| MACRO277-06 |  | Subtidal (6 m) on rock | JX572126 |
| MACRO684-07 |  | Mid intertidal on cobble in freshwater runoff | JX572146 |
| MACRO685-07 |  | Upper mid intertidal pool on rock | JX572036 |
| MACRO686-07 |  | Upper mid intertidal pool on rock | JX571993 |
| MACRO687-07 |  | Upper mid intertidal pool on rock | JX572109 |
| MACRO698-07 |  | Low intertidal pool on rock | JX572137 |
| MACRO699-07 |  | Low intertidal pool on rock | JX572140 |
| MACRO1048-08 |  | Low intertidal pool on rock | JX572133 |
| MACRO701-07 |  | Lower intertidal pools on rock | JX572008 |
| MACRO1417-09 |  | Mid pool on rock | HM891049 |
| MACRO1058-08 |  | Mid pool on rock | JX572071 |
| MACRO463-07 | *Leptonematella fasciculata* (Reinke) P.C. Silva | Subtidal (at 3 m) on *Chorda* (GWS005236) | JX572019 |
| MACRO1375-09 |  | Subtidal (2 m) on *Chaetomorpha* | HM891018 |
| MACRO1012-08 |  | On Drift *Palmaria* (GWS005530) | JX572072 |
| Dictyosiphonaceae |  |  |  |
| MACRO958-08 | *Dictyosiphon foeniculaceus* (Hudson) Greville | Subtidal (1 m) on *Chordaria* | JX572136 |
| MACRO402-06 |  | Mid lower pools on *Chordaria* | JX572081 |
| MACRO395-06 | *Dictyosiphon* sp._1GWS | Subtidal (4 M) on *Chordaria* | JX572013 |
| MACRO403-06 | *Dictyosiphon* sp._3GWS | Mid lower pools on *Fucus* | JX572024 |
| Punctariaceae |  |  |  |
| MACRO1050-08 | *Punctaria* sp._2GWS | Mid intertidal pool on rock | JX572093 |
| MACRO700-07 |  | Mid intertidal pool on rock | JX572083 |
| MACRO1057-08 |  | Low/mid intertidal pool on *Fucus* | JX572122 |
| Scytosiphonaceae |  |  |  |
| MACRO1355-09 | *Petalonia fascia* (O.F. Müller) Kuntze | Mid intertidal pool on cobble | HM891002 |
| MACRO1472-10 |  | Subtidal (3 m) on cobble | HQ990461 |
| MACRO259-06 | *Petalonia filiformis* (Batters) Kuntze | Subtidal (1 m) on rock | JX572033 |
| MACRO260-06 |  | Subtidal (1 m) on rock | JX571986 |
| MACRO396-06 |  | Subtidal (1 m) on rock | JX572125 |
| MACRO261-06 |  | Subtidal (1 m) on rock | JX572106 |
| MACRO614-07 |  | Upper intertidal pool on rock | JX572100 |
| MACRO268-06 |  | Mid intertidal pool on cobble | JX572018 |
| MACRO1448-09 |  | Mid intertidal pool on rock | HM891074 |
| MACRO959-08 | *Scytosiphon canaliculatus* (Setchell & N.L. Gardner) Kogame | Mid intertidal pool on cobble | JX572069 |
| MACRO1046-08 |  | Mid intertidal pool on rock | JX571967 |
| MACRO697-07 |  | Mid intertidal pool on rock | JX572050 |
| MACRO1457-09 |  | Low intertidal pool on rock | HM891080 |
| MACRO1403-09 | *Scytosiphon* sp._1crust | Low intertidal pool on rock | HM891038 |
| Striariaceae |  |  |  |
| MACRO1390-09 | *Stictyosiphon soriferus* (Reinke) Rosenvinge | Subtidal (6 m) on rock | HQ990459 |
| MACRO1016-08 | *Stictyosiphon tortilis* (Ruprecht) Reinke | Subtidal (5 m) on rock | JX572049 |
| MACRO1017-08 |  | Subtidal (1 m) on rock | JX572046 |
| MACRO1010-08 |  | Drift tangled with green in area of freshwater runoff | JX571977 |
| MACRO1019-08 |  | Subtidal (1 m) on rock | JX572113 |
| MACRO1011-08 |  | Subtidal (1 m) on/tangled with *Polysiphonia* (GWS005293) | JX572134 |
| MACRO1020-08 |  | Subtidal (1 m) on rock | JX572002 |
| MACRO1021-08 |  | Subtidal (1 m) on rock | JX572149 |
| MACRO1156-09 |  | Mid pool on rock | HM890865 |
| MACRO1063-08 |  | Mid pool on *Fucus* | JX572099 |
| Fucales |  |  |  |
| Fucaceae |  |  |  |
| MACRO344-06 | *Fucus distichus* Linnaeus | Subtidal (3 m) on rock | EU646733 |
| Laminariales |  |  |  |
| Alariaceae |  |  |  |
| MACRO346-06 | *Alaria esculenta* (Linnaeus) Greville | Drift | JX572060 |
| MACRO352-06 |  | Subtidal (13 m) on rock | JX572012 |
| MACRO1409-09 |  | Subtidal (13 m) on rock | HM891042 |
| MACRO965-08 |  | Subtidal (13 m) on rock | JX572070 |
| MACRO1022-08 |  | Subtidal (12 m) on rock | JX571992 |
| MACRO1034-08 |  | Subtidal (8 m) on rock | JX572086 |
| Chordaceae |  |  |  |
| MACRO256-06 | *Chorda* sp._1*filum* | Subtidal (3 m) on pebble | JX572025 |
| MACRO262-06 |  | Subtidal (1 m) on pebble | JX572032 |
| MACRO265-06 |  | Subtidal (1 m) on pebble | JX572101 |
| MACRO266-06 |  | Mid intertidal pool on rock | JX572143 |
| MACRO267-06 |  | Drift | JX572115 |
| MACRO464-07 |  | Mid intertidal pool on rock | JX572154 |
| MACRO961-08 |  | Subtidal (2 m) on rock | JX572038 |
| MACRO1055-08 |  | Mid pool on rock | JX572092 |
| MACRO1056-08 |  | Mid pool on rock | JX572138 |
| MACRO1061-08 |  | Mid pool on rock | JX572085 |
| Costariaceae |  |  |  |
| MACRO613-07 | *Agarum clathratum* Dumort. | Drift | FJ409116 |
| MACRO1397-09 |  | Subtidal (13 m) on rock | HM891033 |
| MACRO465-07 |  | Subtidal (13 m) on rock | JX572096 |
| MACRO1443-09 |  | Subtidal (12 m) on rock | HM891069 |
| MACRO1024-08 |  | Subtidal (12 m) on rock | JX572034 |
| MACRO1425-09 |  | Subtidal (7 m) on rock | HM891055 |
| MACRO1044-08 |  | Drift | JX572058 |
| Laminariaceae |  |  |  |
| MACRO272-06 | *Laminaria digitata* (Hudson) J.V. Lamouroux | Subtidal (7 m) on rock | FJ409151 |
| MACRO615-07 |  | Subtidal (7 m) on rock | GU097681 |
| MACRO616-07 | *Laminaria solidungula* J. Agardh | Subtidal (6 m) on rock | FJ409166 |
| MACRO283-06 |  | Subtidal (13 m) on rock | FJ409165 |
| MACRO617-07 |  | Subtidal (13 m) on rock | FJ409164 |
| MACRO466-07 |  | Subtidal (13 m) on rock | FJ409163 |
| MACRO964-08 |  | Subtidal (13 m) on rock | GU097713 |
| MACRO1431-09 |  | Subtidal (12 m) on rock | HM891061 |
| MACRO967-08 |  | Subtidal (12 m) on rock | FJ409162 |
| MACRO1435-09 |  | Subtidal (8 m) on rock | HM891063 |
| MACRO1426-09 |  | Drift | HM891056 |
| MACRO264-06 | *Saccharina groenlandica* (Rosenvinge) C.E. Lane, Mayes, Druehl & G.W. Saunders | Drift | GU097739 |
| MACRO963-08 |  | Subtidal (13 m) on rock | GU097737 |
| MACRO281-06 |  | Subtidal (13 m) on rock | GU097743 |
| MACRO282-06 |  | Subtidal (13 m) on rock | GU097740 |
| MACRO1033-08 |  | Subtidal (8 m) on rock | GU097736 |
| MACRO1413-09 |  | Subtidal (8 m) on rock | HM891045 |
| MACRO695-07 |  | Drift | GU097745 |
| MACRO1436-09 |  | Drift | HM891064 |
| MACRO1381-09 |  | Subtidal (10 m) on rock | HM891022 |
| MACRO1392-09 |  | Subtidal (10 m) on rock | HM891028 |
| MACRO1428-09 |  | Subtidal (10 m) on rock | HM891058 |
| MACRO612-07 | *Saccharina latissima* (Linnaeus) C.E. Lane, Mayes, Druehl & G.W. Saunders | Subtidal (6 m) on rock | GU097792 |
| MACRO255-06 |  | Subtidal (6 m) on rock | GU097797 |
| MACRO257-06 |  | Drift | GU097802 |
| MACRO270-06 |  | Subtidal (6 m) on rock | GU097812 |
| MACRO271-06 |  | Subtidal (6 m) on rock | GU097816 |
| MACRO285-06 |  | Subtidal (13 m) on rock | GU097805 |
| MACRO966-08 |  | Subtidal (12 m) on rock | FJ409199 |
| MACRO1023-08 |  | Subtidal (12 m) on rock | GU097762 |
| MACRO1025-08 |  | Subtidal (12 m) on rock | GU097790 |
| MACRO1026-08 |  | Drift | GU097788 |
| MACRO1027-08 |  | Drift | GU097785 |
| MACRO1030-08 |  | Drift | GU097795 |
| MACRO1031-08 |  | Drift | GU097791 |
| MACRO1032-08 |  | Drift | GU097761 |
| MACRO1035-08 |  | Subtidal (6 m) on rock | GU097760 |
| MACRO1036-08 |  | Subtidal (6 m) on rock | GU097759 |
| MACRO1037-08 |  | Subtidal (6 m) on rock | GU097758 |
| MACRO1038-08 |  | Subtidal (6 m) on rock | GU097757 |
| MACRO1039-08 |  | Subtidal (6 m) on mussel | GU097793 |
| MACRO1040-08 |  | Subtidal (6 m) on mussel | GU097787 |
| MACRO690-07 |  | Drift | GU097806 |
| MACRO691-07 |  | Drift | GU097810 |
| MACRO1041-08 |  | Drift | GU097796 |
| MACRO692-07 |  | Drift | GU097818 |
| MACRO693-07 |  | Drift | GU097817 |
| MACRO694-07 |  | Drift | GU097819 |
| MACRO1092-09 |  | Drift | GU097754 |
| MACRO1414-09 |  | Drift | HM891046 |
| MACRO1042-08 |  | Drift | GU097756 |
| MACRO1043-08 |  | Drift | GU097755 |
| MACRO1415-09 |  | Drift | HM891047 |
| MACRO1427-09 |  | Drift | HM891057 |
| MACRO1437-09 |  | Drift | HM891065 |
| MACRO1404-09 |  | Subtidal (10 m) on rock | HM891039 |
| MACRO1416-09 |  | Subtidal (10 m) on rock | HM891048 |
| Sphacelariales |  |  |  |
| Sphacelariaceae |  |  |  |
| MACRO1018-08 | *Sphacelaria radicans* (Dillwyn) Harvey | Subtidal turf (1 m) on cobble | JX572040 |
| MACRO394-06 | *Sphacelaria rigidula* Kützing | Subtidal (3 m) on algae | JX572010 |
| MACRO1013-08 |  | Drift on algae | JX572059 |
| Tilopteridales |  |  |  |
| Halosiphonaceae |  |  |  |
| MACRO275-06 | *Halosiphon* sp._2*tomentosus* (Lyngbye) Jaasund | Subtidal (2 m) on rock | JX571976 |
| Tilopteraceae |  |  |  |
| MACRO1155-09 | *Haplospora globosa* Kjellman | Subtidal (7 m) on rock | HM890864 |
| Florideophyceae |  |  |  |
| Ahnfeltiales |  |  |  |
| Ahnfeltiaceae |  |  |  |
| ABMMC2102-08 | *Ahnfeltia borealis* D. Milstein & G.W. Saunders | Subtidal (5 m) on rock | JN113243 |
| ABMMC1762-07 |  | Subtidal (4 m) on rock | JN113245 |
| ABMMC1758-07 |  | Subtidal (6 m) on rock | JN113241 |
| ABMMC1759-07 |  | Subtidal (6 m) on rock | JN113248 |
| ABMMC1760-07 |  | Drift | JN113247 |
| ABMMC1761-07 |  | Low intertidal pool on rock | JN113246 |
| ABMMC2549-08 |  | Low intertidal pool on rock | JN113244 |
| ABMMC1757-07 | *Ahnfeltia plicata* (Hudson) Fries | Drift | JN113199 |
| ABMMC1576-07 |  | Drift | JN113200 |
| Corallinales |  |  |  |
| Hapalidiaceae |  |  |  |
| ABMMC9449-10 | *Lithothamnion glaciale* Kjellman | Low intertidal pool on rock | HM918759 |
| ABMMC2412-08 | *Phymatolithon lenormandii* (Areschoug) W.H. Adey | Subtidal (13 m) on cobble | JX571978 |
| ABMMC9439-10 |  | Subtidal (13 m) on cobble | HM918752 |
| ABMMC9440-10 |  | Subtidal (13 m) on cobble | JX572005 |
| Acrochaetiales |  |  |  |
| Acrochaetiaceae |  |  |  |
| ABMMC2864-08 | *Acrochaetium* sp. | Subtidal (12 m) on *Rhodomela* (GWS005411) | JX571988 |
| Palmariales |  |  |  |
| Palmariaceae |  |  |  |
| ABMMC2113-08 | *Devaleraea ramentacea* (Linnaeus) Guiry | Subtidal (6 m) on rock | GU224094 |
| ABMMC2548-08 |  | Subtidal (6 m) on rock | JX571995 |
| ABMMC1763-07 |  | Subtidal (6 m) on rock | JX572120 |
| ABMMC2803-08 | *Palmaria palmata* (Linnaeus) Kuntze | Subtidal (5 m) on rock | JX572074 |
| ABMMC2863-08 |  | Subtidal (6 m) on rock | JX572075 |
| ABMMC2867-08 |  | Subtidal (6 m) on rock | JX572004 |
| ABMMC2114-08 |  | Subtidal (6 m) on rock | JX572057 |
| ABMMC2116-08 |  | Subtidal (6 m) on rock | JX572067 |
| ABMMC2805-08 |  | Drift | JX572132 |
| ABMMC2118-08 |  | Drift on *Palmaria* (GWS005530) | GU224116 |
| ABMMC2119-08 | *Rhodophysema kjellmanii* G.W. Saunders & Clayden | Drift on *Palmaria* (GWS005530) | GU224121 |
| ABMMC9445-10 |  | Drift on *Palmaria* (GWS005530) | HM918757 |
| Ceramiales |  |  |  |
| Ceramiaceae |  |  |  |
| ABMMC3580-08 | *Scagelia* sp. | Subtidal (6 m) on *Rhodomela* | JX571996 |
| ABMMC3581-08 |  | Subtidal (6 m) on *Rhodomela* | JX572068 |
| ABMMC1328-07 |  | Subtidal (5 m) on *Ahnfeltia* | JX572103 |
| ABMMC1679-07 |  | On drift *Sphacelaria* | JX572011 |
| ABMMC1764-07 |  | Subtidal (1 m) on *Phycodrys* (GWS005302) | JX571983 |
| ABMMC3582-08 |  | Subtidal (6 m) on algae | JX572157 |
| ABMMC1765-07 |  | Subtidal (13 m) on *Desmarestia* | JX572114 |
| ABMMC2107-08 |  | Subtidal (12 m) on rock | JX571972 |
| ABMMC2778-08 |  | Subtidal (12 m) on rock | JX572079 |
| ABMMC4430-09 |  | Subtidal (18 m) on inverts | XXXXXX |
| ABMMC2779-08 |  | Drift | JX572044 |
| ABMMC3584-08 |  | Subtidal (9 m) on *Rhodomela* | JX572020 |
| ABMMC2780-08 |  | Subtidal (6 m) on *Ahnfeltia* | JX572017 |
| ABMMC2782-08 |  | Subtidal (6 m) on *Phycodrys* | JX572016 |
| ABMMC3585-08 |  | Subtidal (6 m) on *Polysiphonia* | JX572141 |
| ABMMC1766-07 |  | Drift on *Polysiphonia* GWS005536 | JX572082 |
| ABMMC3586-08 |  | Subtidal (4 m) on *Odonthalia* | JX572105 |
| ABMMC3587-08 |  | Subtidal (4 m) on *Odonthalia* | JX572053 |
| Delesseriaceae |  |  |  |
| ABMMC5996-09 | *Phycodrys fimbriata* (Kuntze) Kylin | Subtidal (6 m) on rock | HM916620 |
| ABMMC6008-09 |  | Subtidal (6 m) on rock | HM916630 |
| ABMMC6019-09 |  | Subtidal (6 m) on rock | JX572073 |
| ABMMC5937-09 |  | Subtidal (1 m) on rock | HM916572 |
| ABMMC5949-09 |  | Subtidal (1 m) on *Fucus* | HM916581 |
| ABMMC5961-09 |  | Subtidal (6 m) on rock | HM916591 |
| ABMMC5973-09 |  | Subtidal (12 m) on rock | HM916602 |
| ABMMC5985-09 |  | Drift | HM916611 |
| Rhodomelaceae |  |  |  |
| ABMMC1324-07 | *Odonthalia dentata* (Linnaeus) Lyngbye | Subtidal (6 m) on *Rhodomela* | JX572001 |
| ABMMC1325-07 |  | Subtidal (6 m) on *Rhodomela* | JX571971 |
| ABMMC1329-07 |  | Drift | JX572108 |
| ABMMC1330-07 |  | Subtidal (6 m) on rock | JX572142 |
| ABMMC1767-07 |  | Subtidal (12 m) on rock | JX571960 |
| ABMMC9444-10 |  | Subtidal (6 m) on rock | HM918756 |
| ABMMC1768-07 |  | Subtidal (10 m) on rock | JX572084 |
| ABMMC2103-08 | *Polysiphonia arctica* J. Agardh | Subtidal (5 m) on *Ahnfeltia* | JX572047 |
| ABMMC9437-10 |  | Subtidal (6 m) on rock | HM918751 |
| ABMMC9441-10 |  | Subtidal (18 m) on inverts | HM918753 |
| ABMMC9442-10 |  | Drift | HM918754 |
| ABMMC2115-08 |  | Subtidal (6 m) on *Ahnfeltia* | JX571980 |
| ABMMC9446-10 |  | Drift | JX572043 |
| ABMMC9431-10 | *Polysiphonia* sp._*1stricta* (Dillwyn) Greville | Subtidal (1 m) on *Fucus* | HM918749 |
| ABMMC9432-10 |  | Subtidal (1 m) on rock | JX571973 |
| ABMMC9435-10 |  | Subtidal (1 m) on rock | HM918750 |
| ABMMC9465-10 |  | Mid pool on *Fucus* | HM918770 |
| ABMMC9433-10 | *Polysiphonia* sp._*3stricta* (Dillwyn) Greville | Subtidal (1 m) on mussel | HQ919612 |
| ABMMC9468-10 |  | Mid pool in sand | HQ919613 |
| ABMMC2208-08 | *Rhodomela confervoides* (Hudson) P.C. Silva | Drift | JX572076 |
| ABMMC1770-07 | *Rhodomela lycopodioides* (Linnaeus) C. Agardh | Subtidal (5 m) on rock | JX572062 |
| ABMMC1780-07 |  | Subtidal (1 m) on rock | JX572066 |
| ABMMC2110-08 |  | Upper intertidal pool on cobble | JX571966 |
| ABMMC2865-08 |  | Upper intertidal pool on cobble | JX572153 |
| ABMMC2866-08 |  | Low intertidal pool on *Fucus* | JX572063 |
| ABMMC2111-08 |  | Drift | JX571984 |
| ABMMC2112-08 |  | Upper intertidal pool on cobble | JX572147 |
| ABMMC1771-07 |  | Subtidal (6 m) on rock | JX571985 |
| ABMMC1772-07 |  | Low intertidal pool on rock | JX572027 |
| ABMMC1773-07 |  | Low intertidal pool on rock | JX572055 |
| ABMMC1774-07 |  | Low intertidal on mussel | JX572061 |
| ABMMC1326-07 | *Rhodomela lycopodioides* f. *flagellaris* Kjellman | Subtidal (6 m) on rock | JX571959 |
| ABMMC9428-10 |  | Subtidal (6 m) on rock | HM918748 |
| ABMMC1327-07 |  | Subtidal (6 m) on rock | JX572054 |
| ABMMC1331-07 |  | Subtidal (6 m) on rock | JX572030 |
| ABMMC1332-07 |  | Subtidal (6 m) on rock | JX572094 |
| ABMMC1333-07 |  | Subtidal (13 m) on rock | JX572014 |
| ABMMC2804-08 |  | Subtidal (12 m) on rock | JX572156 |
| ABMMC2109-08 |  | Subtidal (12 m) on rock | JX571974 |
| ABMMC1775-07 |  | Subtidal (9 m) on rock | JX572121 |
| ABMMC2117-08 |  | Drift | JX571975 |
| ABMMC9447-10 |  | Drift | HM918758 |
| ABMMC2120-08 |  | Subtidal (4 m) on rock | JX572135 |
| ABMMC1776-07 |  | Subtidal (4 m) on rock | JX572127 |
| ABMMC1206-07 | *Rhodomela virgata* Kjellman | Subtidal (5 m) on rock | JX572089 |
| ABMMC1063-06 |  | Subtidal (6 m) on rock | JX572150 |
| ABMMC1777-07 |  | Subtidal (6 m) on rock | JX571991 |
| ABMMC1778-07 |  | Subtidal (5 m) on rock | JX572119 |
| ABMMC1779-07 |  | Subtidal (5 m) on rock | JX572116 |
| ABMMC478-06 |  | Subtidal (1 m) on *Fucus* | JX572118 |
| ABMMC1207-07 |  | Subtidal (1 m) on rock | JX571961 |
| ABMMC2105-08 |  | Drift on *Coccotylus* | JX572078 |
| ABMMC1781-07 |  | Subtidal (6 m) on rock | JX571987 |
| ABMMC1782-07 |  | Subtidal (6 m) on rock | JX572128 |
| ABMMC1783-07 |  | Subtidal (6 m) on rock | JX571981 |
| ABMMC2108-08 |  | Subtidal (12 m) on rock | JX572152 |
| ABMMC1784-07 |  | Subtidal (12 m) on rock | JX572026 |
| ABMMC1785-07 |  | Subtidal (12 m) on rock | JX572037 |
| ABMMC1786-07 |  | Drift | JX572131 |
| ABMMC1787-07 |  | Drift | JX571969 |
| ABMMC1788-07 |  | Low intertidal pool on rock | JX572041 |
| ABMMC1789-07 |  | Low intertidal pool on *Fucus* | JX571997 |
| ABMMC9443-10 |  | Drift | HM918755 |
| ABMMC1790-07 |  | Subtidal (9 m) on rock | JX572095 |
| ABMMC1791-07 |  | Subtidal (6 m) on rock | JX572028 |
| ABMMC2207-08 |  | Subtidal (6 m) on mussel | JX572039 |
| ABMMC1792-07 |  | Low intertidal pool on mussel | JX572145 |
| ABMMC1793-07 |  | Mid intertidal pool on *Fucus* | JX572148 |
| ABMMC1794-07 |  | Low intertidal pool on mussel | JX571968 |
| ABMMC1795-07 |  | Subtidal (5 m) on rock | JX572009 |
| ABMMC9466-10 |  | Mid pool on rock | HM918771 |
| Wrangeliaceae |  |  |  |
| ABMMC3583-08 | *Ptilota gunneri* P.C. Silva, Maggs & L.M. Irvine | Drift | JX571999 |
| Gigartinales |  |  |  |
| Dumontiaceae |  |  |  |
| ABMMC1367-07 | *Dilsea socialis* (Postels & Ruprecht) Perestenko | Subtidal (6 m) on rock | EU189281 |
| ABMMC1368-07 |  | Subtidal (6 m) on rock | EU189282 |
| ABMMC1528-07 |  | Subtidal (6 m) on rock | EU189284 |
| ABMMC995-06 |  | Drift | EU189280 |
| ABMMC1369-07 |  | Subtidal (6 m) on rock | EU189283 |
| ABMMC2106-08 |  | Subtidal (13 m) on rock | JX572031 |
| ABMMC1798-07 |  | Subtidal (12 m) on rock | JX571982 |
| ABMMC1799-07 |  | Subtidal (12 m) on rock | JX572056 |
| ABMMC1800-07 |  | Subtidal (6 m) on rock | JX572123 |
| ABMMC1801-07 |  | Drift | JX572051 |
| Phyllophoraceae |  |  |  |
| ABMMC1131-06 | *Coccotylus brodiei* (Turner) Kützing | Subtidal (5 m) on mussel | GQ380038 |
| ABMMC1130-06 | *Coccotylus truncatus* (Pallas) Wynne & Heine | Subtidal (6 m) on rock | GQ380071 |
| ABMMC9429-10 |  | Subtidal (6 m) on rock | JX572090 |
| ABMMC2104-08 |  | Subtidal (5 m) on rock | GQ380075 |
| ABMMC1132-06 |  | Subtidal (6 m) on rock | GQ380069 |
| ABMMC577-06 |  | Subtidal (6 m) on rock | GQ380076 |
| ABMMC2205-08 |  | Subtidal (12 m) on rock | GQ380077 |
| ABMMC2206-08 |  | Subtidal (6 m) on rock | GQ380073 |
| ABMMC1813-07 |  | Drift | GQ380086 |
| ABMMC1814-07 |  | Subtidal (3 m) on rock | GQ380087 |

^1^Ulvophyceae marker *tufA* only.

^2^Ulvophyceae marker *tufA* and *rbc*L-3P.

^3^Ulvophyceae marker *rbc*L-3P only.
